# *In-vivo* monitoring of infectious diseases in living animals using bioluminescence imaging

**DOI:** 10.1080/21505594.2017.1371897

**Published:** 2017-12-08

**Authors:** Pinar Avci, Mahdi Karimi, Magesh Sadasivam, Wanessa C. Antunes-Melo, Elisa Carrasco, Michael R. Hamblin

**Affiliations:** a Wellman Center for Photomedicine, Massachusetts General Hospital, Boston, MA, USA; b Department of Dermatology, Harvard Medical School, Boston, MA, USA; c Department of Medical Nanotechnology, School of Advanced Technologies in Medicine, Iran University of Medical Sciences, Tehran, Iran; d Cellular and Molecular Research Center, Iran University of Medical Sciences, Tehran, Iran; e Amity Institute of Nanotechnology, Amity University Uttar Pradesh, Noida, India; f University of Sao Paulo, Sao Carlos-SP, Brazil; g Department of Biosciences, Durham University, Durham, United Kingdom; h Harvard-MIT Division of Health Sciences and Technology, Cambridge, MA, USA

**Keywords:** Bioluminescence imaging, infectious disease pathogenesis, luciferase, genetic engineering, bacteria, fungi, viruses, parasites

## Abstract

Traditional methods of localizing and quantifying the presence of pathogenic microorganisms in living experimental animal models of infections have mostly relied on sacrificing the animals, dissociating the tissue and counting the number of colony forming units. However, the discovery of several varieties of the light producing enzyme, luciferase, and the genetic engineering of bacteria, fungi, parasites and mice to make them emit light, either after administration of the luciferase substrate, or in the case of the bacterial *lux* operon without any exogenous substrate, has provided a new alternative. Dedicated bioluminescence imaging (BLI) cameras can record the light emitted from living animals in real time allowing non-invasive, longitudinal monitoring of the anatomical location and growth of infectious microorganisms as measured by strength of the BLI signal. BLI technology has been used to follow bacterial infections in traumatic skin wounds and burns, osteomyelitis, infections in intestines, Mycobacterial infections, otitis media, lung infections, biofilm and endodontic infections and meningitis. Fungi that have been engineered to be bioluminescent have been used to study infections caused by yeasts (Candida) and by filamentous fungi. Parasitic infections caused by malaria, Leishmania, trypanosomes and toxoplasma have all been monitored by BLI. Viruses such as vaccinia, herpes simplex, hepatitis B and C and influenza, have been studied using BLI. This rapidly growing technology is expected to continue to provide much useful information, while drastically reducing the numbers of animals needed in experimental studies.

## Introduction to bioluminescent organisms

Bioluminescence is used by various organisms, including microorganisms, for various purposes including communication, reproduction, and defense from predators, and is defined as the enzymatic production of visible light from cells. The use of photoactive proteins in biology and medicine commenced with the original isolation and modification of green fluorescent protein (GFP) as well as the transfection of *Escherichia coli (E. coli)* with the GFP gene, for which Chalfie, Shimomura and Tsien won the Nobel Prize in Chemistry in 2008.^1^ The gene sequence for firefly luciferase and its mechanism of action was determined by Marlene Deluca starting in the 1970s.^2^ These two original types of light emitting proteins have led to an explosion of interest in bioluminescence in molecular biology and biomedical sciences, which has grown beyond the initial use as molecular probes for microscopic studies.

**Figure 1.**
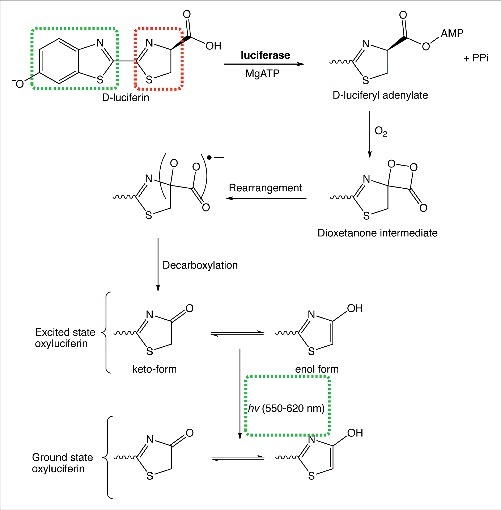
Chemical reactions leading to light emission from D-luciferin and firefly luciferase.

Firefly luciferase is an oxidative enzyme that generates light in a classical and well-understood multistep mechanism (Fig. 1). In eukaryotes, D-luciferin is initially adenylated by Mg-ATP, generating D-luciferyl-adenylate and pyrophosphate. D-luciferyl-adenylate is then oxidized in the presence of an equivalent of molecular oxygen (O_2_) yielding a highly strained dioxetenone ring (in red), which is relieved by a homolytic O-O bond cleavage. Decarboxylation not only relieves the instability of the radical adduct, but also generates excited oxyluciferin. Oxyluciferin (aromatic in the enol form) tautomerizes with the keto form. Remarkably, both the enol and keto forms of excited oxyluciferin are capable of relaxing back to ground state oxyluciferin with the consequent emission of a visible photon.^3^ The light emitted in the process ranges anywhere from 550 nm (lime green) to 620 nm (red) and the reason for variations in color has yet to be unambiguously identified.^4^ To date, variation in the excited state oxyluciferin emission wavelength is thought to be a consequence of keto/enol population densities, the torsional angle between thiazole and benzothiazole (red and green respectively in Figure 1), or the microenvironment in which the decay process occurs.^5^

Luciferase enzymes have been found to be expressed in a wide range of different life forms.^6^ It has been estimated using phylogenetic analysis that luciferase systems may have arisen from more than 30 independent evolutionary origins.^7^ In addition to the beetle luciferase enzymes, found in fireflies and click beetles, marine organisms and bacteria have provided rich sources of luciferase systems. Bioluminescence is also found in dinoflagellates^8^ and some fungi.^9^ In some marine organisms such as Renilla, the luciferase is closely coupled to a fluorescent protein such as GFP to red shift the emission from the blue to the green spectrum. Due to the increasing demand for these light-emitting systems both for use in luciferase reporter assays and for bioluminescence imaging (BLI), molecular biologists and genetic engineers have carried out numerous modification and optimization procedures on the amino-acid sequences of these proteins. Table 1 shows the different luciferase enzymes that have become important in bioluminescence imaging.

**Table 1.** Variants of luciferase enzyme together with their substrates and wavelengths commonly used in bioluminescence imaging (BLI).

Luciferase	Susbtrate	Emission peak	Comments	Reference
Firefly (*Photinus pyralis*) FLuc	D-luciferin	612 nm	Requires ATP; substrate has to penetrate cells; optimized for expression in mammalian cells; versions with increased thermostability availability	^199^
Click beetle (*Pyrophorus plagiophthalamus*) green CBGr99		611 nm		^200^
Click beetle red CBred		544 nm		^201^
Sea pansy (*Renilla reniformis*) RLuc	Coelenterazine	480 nm	Does not require ATP; substrate has to penetrate cells; commonly used for mammalian cells;	^202^
Renilla mutant Rluc 8.6–535		535 nm		^203^
Mesopelagic copepod (*Gaussia princeps)* GLuc		480 nm	Secreted enzyme; membrane localized versions available	^204^
NanoLuc (mutant *Oplophorus gracilirostris*) NLuc	Furimazine		Small (19.1 kDa); enhanced stability; >150-fold increase in luminescence.	^14^
Bacterial luciferase (*Photorhabdus luminescens)* lux	Endogenous reduced flavin mononucleotide	490 nm	Lux operon (*luxCDABE*) for Gram-negative (*luxABCDE*) for Gram-positive bacteria; *luxAB* encodes 2 subunits of luciferase; l*uxCDE* encode fatty acid reductase	^205^
					Long chain aldehyde (e.g. nonanal)

For BLI in animals the following advantages and disadvantages must be taken into account. The peak wavelength of the emission is important for efficient detection by imaging systems because red light is significantly less absorbed by endogenous chromophores and is also significantly less scattered by tissue. All luciferases are oxidizing enzymes and need the presence of significant amounts of O_2_ to function optimally, so their activity in acutely hypoxic tissues may be compromised. Moreover beetle luciferases also need cellular ATP to function, so ATP availability may be a limiting factor. For systems that need administration of exogenous luciferase substrates, the penetration of the substrate molecule into the cells is important and the pharmacokinetics and biodistribution of the substrate must also be taken into account. Besides both D-luciferin and coelenterazine have been found to be substrates of multi-dug efflux transporters such as ABCG2 and p-glycoprotein.^10,11^ The Gaussia luciferase (Gluc) is secreted from the cells, and this will increase the background signal in *in-vivo* imaging.^12^ The bacterial luciferase operon should be stably integrated into the bacterial chromosome using a transposon to avoid the loss of plasmids.^13^ The precise promoter employed in the genetic construct also has a major effect on the efficiency of bioluminescence production.^6^ The recent introduction of NanoLuc (NLuc) has caused some interest.^14^ A luciferase enzyme was isolated from the deep-sea shrimp *Oplophorus gracilirostris*, and underwent three rounds of mutagenesis to produce the novel NLuc system. This enzyme is small (only 19.1 kDa), and its specific activity is over 150-fold higher than FLuc and RLuc. Its novel substrate, furimazine, provides additional possibilities to carry out multiplexed imaging studies. One of the most exciting applications of bioluminescence (and the topic of this review) is the use of BLI to model host/pathogen interactions and track disease progress. This invaluable scientific technology relies on the engineering of either the host or the pathogen to express luciferase enzymes, rather than GFP.^15^ BLI for infectious diseases is surprisingly similar to the observation of “glowing wounds” (termed “Angel's Glow”) that was seen in injured soldiers during the American Civil War. These infected wounds were not only non-lethal to the soldiers, but field surgeons observed that wounds that happened to display visible luminescence actually promoted patient survival. Nowadays, it is understood that these “glowing wounds” were a consequence of infection by the gamma-proteobacteria *Photorhabdus luminescens* (previously called *Xenorhabdus luminescens*) native to the gut of nematodes. Angel's Glow is due to the bacterial luciferase system of *P. luminescens* and the enhanced patient survival was due to production of antibiotics by *P. luminescens* which prevented growth of otherwise more lethal wound pathogens.

In contrast to the aforementioned luciferase systems of eukaryotes, the prokaryotic bioluminescence that is catalyzed by a different luciferase is dependent on the oxidation of long-chain aldehydes reacting with reduced flavin mononucleotide in the presence of oxygen, yielding the oxidized flavin, a long-chain fatty acid, and light.^16^ While the mechanism of light production differs markedly between prokaryotes and eukaryotes in terms of substrate specificity, the point which is worth noting, is that the different luciferase enzymes are highly specialized and capable of facilitating several distinct chemical processes that result in light production.

The *lux* operon found in *P. luminescens* is convenient for BLI purposes in that it contains both the genes for the synthesis of luciferase and for the synthesis of the aldehyde substrate,^3^ so no additional substrate needs to be added. On the other hand, the use of the *P. pyralis* luciferase and marine luciferase enzymes is less desirable in infections in that exogenous D-luciferin or coelenterazine must be administered rather than the substrate being endogenously synthesized in cells. Accordingly, in 1995 Contag *et al*^17^ successfully transferred the *P. luminescens lux* operon (*luxCDABE*) to the Gram-negative enteropathogen *Salmonella typhimurium* (*S. typhimurium*) and since then successful transfection has been carried out in a plethora of different microorganisms. It was found to be necessary to use a modified *P. luminescens lux* transposon plasmid pAUL-Atn4001 luxABCDE-Kmr that had been specifically tailored for Gram-positive bacteria.^18^ This is because the *P. luminescens lux CDABE* operon (that functions well in Gram-negative bacteria) is not translated in Gram-positive bacteria, as these organisms do not have the correct ribosome-binding sites in the mRNA sequences. By reorganizing the gene order in the cassette to ABCDE instead of CDABE and inserting a Gram-positive BBBFGD32 ribosome-binding site upstream of all 5 genes contained within the operon, Gram-positive bacteria could then be stably transformed.

**Figure 2.**
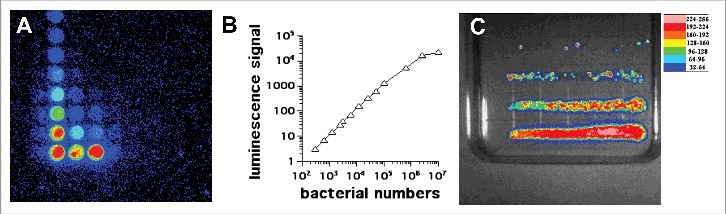
In vitro studies with *P. aeruginosa* XEN41. (A) Serial dilutions in a 96 well plate imaged by BLI. (B) Correlation between luminescence signal and number of CFU. (C) Colonies formed by streaking bacteria on an agar plate according to Jett et al.^34^

The principle behind the use of BLI for modeling and monitoring infectious diseases is simple yet extremely useful. Provided an animal or model organism is solely infected with a microbial strain that expresses the bacterial luciferase enzyme system, the light production (typically measured at 490 nm for the *P. luminescens* variant) is proportional to the microbial concentration. With appropriate in-vitro calibration, not only is qualitative information derived but quantitative microbial load estimation may also be made. The *in-vitro* and *in-vivo* correlations are discussed below. To date, the relationship between detected luminescence and microbial load concentration has been used in BLI monitoring of infections caused by the Gram-negative bacteria, *E. coli*,^19^ *Citrobacter*,^20^ the Gram-positive bacteria *Staphyloccocus aureus* (*S. aureus*) (methicillin-intermediate and resistant isolates),^18^ and *Streptococcus pneumoniae* (*S. pneumoniae*),^21^ mycobacteria,^22^ *Candida albicans* (*C. albicans*)^23,24,25,26^ and even the filamentous fungi *Aspergillus fumigatus* (*A. fumigatus*).^27^ Fig. 2B shows a correlation plot using *Pseudomonas aeruginosa *(*P. aeruginosa*) Xen41.

BLI provides a number of advantages that can be used to provide information about the dynamics of the infectious processes. Many animal models of human biology and diseases have been investigated successfully using BLI. Recombinant strains of bacteria expressing luciferase, have reduced the need to sacrifice animals at different time-points, so each animal can be used as its own control over the length of the experiment, and overcoming the problem of animal-to–animal variation if groups of animals are sacrificed at different time-points. Animal studies using these bioluminescent strains have provided information via qualitative and quantitative analysis of the microbial load, and have identified progression or migration to previously unknown sites in the body. Many research groups have employed BLI as advantageous technique to monitor the effectiveness of antimicrobial techniques in a variety of animal models of infections caused by different bioluminescent pathogens. These methods have also been validated in mouse models of infected wounds,^28^ burns,^29^ soft tissue infections,^30^ and in dentistry, for endodontic treatment of both Gram-positive and Gram-negative bacteria.^31^

In this paper, we will review *in-vivo* monitoring of infectious diseases in living animals using BLI for bacterial infections in dermal wounds (burns, abrasions, soft tissue and surgical sites), internal bacterial infections (biofilms, endodontics, meningitis, otitis, osteomyelitis, *Salmonella, Mycobacteria* and lung infections), fungal (*Candida, Aspergillus*), eukaryotic parasitic infections (*Plasmodium, Leishmania, Trypanosomes, Toxoplasma*) and viral infections.

## Correlation of Bioluminescence signal of microorganisms with colony forming units

### *In-vitro* correlation

The emission of light from bioluminescent cells isline numbers usually measured by a luminometer either in tube format or in a 96-well luminescence plate format (Fig. 2A) for *P. aeruginosa* Xen41. The lowest number of cells that can be detected depends on the sensitivity of the photomultiplier tube (PMT) involved, but has been reported to be as low as 200 CFU (colony forming units) for bacteria with lux^32^ and 1000 CFU for Candida with GLuc.^33^ The highest number of cells that can be reliably detected is again determined by the saturation point of the PMT, as the linear response is limited at some point. Moreover, it is possible that at very high cell densities, neighboring cells will absorb some of the emitted light and therefore prevent it reaching the PMT. Nevertheless, the signal of bioluminescence vs CFU is linear over several orders of magnitude Fig. 2C shows a serial dilution of bioluminescent bacteria streaked on an agar plate by the method of Jett et al.^34^

### *In-vivo* correlation

The bioluminescence signal from infections in small animals or from model organisms is usually imaged in a highly sensitive CCD camera. These cameras can either be based on an image intensifier attached to the CCD, or on a cooled back-lit CCD camera. The company Xenogen Inc (Alameida, CA; now part of Perkin-Elmer) was instrumental in popularizng this technique in laboratories around the world. Xenogen manufacture a series of IVIS *in-vivo* imaging systems that include bioluminescence along with other modalities. They were also responsible for the genetic engineering of a number microbial strains and cancer cells that stably express various forms of luciferase. Many studies have correlated BLI studies with numbers of CFU determined by sacrificing the animals, removing the tissue, weighing it and then homogenizing the tissue samples in such a way that serial dilutions can allow CFU to be enumerated.

## Animal models of bacterial infectious disease using BLI

### External traumatic skin infection models

External traumatic skin injuries such as surgical wounds, burns, and traumatic abrasions and lacerations result in damage to many structures and cell layers and are frequently complicated by infection leading to prolonged healing. Animal models have been used to study a wide range of different traumatic wound infections and for testing new anti-microbial strategies.^35^ Studies have been carried out that have varied in the animal species used, the strains of microorganisms applied, the number of CFU applied, size of the wounds etc.^36^ Dermal wounds such as excisions result in damage to many structures and cell layers, whereas skin abrasions are wounds where the upper layer of the skin comprising the epidermis has been rubbed off or torn off the and there may also be partial damage to the dermis down to the subcutaneous layer. These external traumatic skin wounds are frequently complicated by infection resulting in prolonged healing Table 2 shows a summary of representative animal models of dermal abrasions, excisional wounds and burn infections that have been monitored by BLI using bioluminescent microorganisms.

**Table 2.** Summary of representative external traumatic wound infection models monitored by bioluminescent imaging (BLI).

Wound model	Host animal species	Bioluminescent microorganism	Methods used to produced external traumatic wounds	Study findings/Treatment	Ref
Dermal needle-scratch	BALB/c mice	Methicillin-resistant *S. aureus* (MRSA)	Mice pre-treated with cyclophosphamide. Skin needle scratch abrasion wounds created on the dorsal surfaces	PDT mediated by PEI-ce6 conjugate + red light. Treated wounds healed faster	^41^
Dermal abrasion	BALB/c mice	MRSA	Abrasion wounds made using a needle by creating orthogonally crossed scratch lines. Bacterial suspension containing 10^8^ CFU of bioluminescent MRSA inoculated on each scratched area	PDT using a phthalocyanine derivative and toluidine blue with red light reduced MRSA signal and stimulated wound healing	^18^
Dermal abrasion	BALB/c mice	*C. albicans*	Scalpel blade is used to scrape the superficial skin until a reddened area appears and then the area is inoculated with bioluminescent *C. albicans*	PDT using phenothiazinium salts and red light	^42^
Dermal excision	Male BALB/c mice	*E. coli*	Full-thickness transdermal excisional wounds created on dorsal surface	Antimicrobial PDT with pL-ce6 conjugate and red light	^28^
Burn wounds	Male BALB/c mice	*Acinetobacter baumannii*	Full-thickness (3^rd^ degree) burn wounds created on dorsal surface of mice	Pulsed electric field (PEF) applied externally	^206^
Burn wounds	Male BALB/c mice	*S. aureus*,	Third-degree dermal burn wounds	Antimicrobial PDT using decacationic monoadducts and bisadducts of^70^ fullerene	^207^
		*A. baumannii*			
		*E. coli*			
Burn wounds	Female BALB/c mice	*C.Pseudomonas aeruginosa*	Full-thickness dermal burns	Blue light (415 nm) treatment offered safe and effective therapy against *P. aerugiosa* infected burn wounds	^208^
Burn wounds	Female BALB/c mice	*C. albicans*	Third degree burn wounds were infected with fungal inoculum	Efficacy of UVC light (254 nm) treatment against *C. albicans* infection monitored by BLI	^209^
Burn wounds	Female BALB/c mice	*S. aureus*	Third-degree burn wounds were infected with *S. aureus*	Antimicrobial PDT mediated by meso-mono-phenyl-tri(N-methyl-4-pyridyl)-porphyrin (PTMPP) was monitored by BLI to treat burn wounds.	^210^
Dermal abrasion and burn wounds	Female BALB/c mice	*A. baumannii and*	Dermal abrasion and full-thickness burn created and inoculated with bioluminescent multi-drug resistant *A. baumannii* isolated from battle-field soldier wounds	Efficacy of UVC light against combat-related wound infection with A. baumannii , monitored by BLI	^211^
		*C. albicans*			

**Figure 3.**
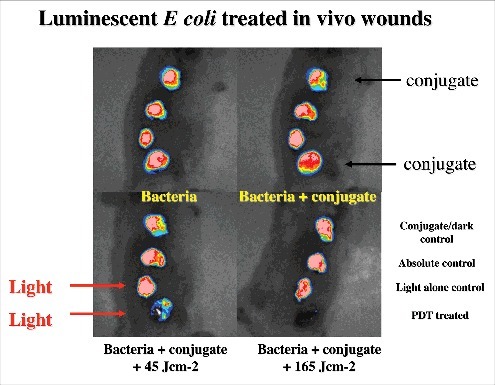
BLI of a mouse model of excisional wounds infected with non-pathogenic *E. coli* and treated with PDT. Figure adapted from data in.^28^

The Hamblin laboratory has developed a series of mouse models of infections viz. excisional-type wounds, scratch wounds and abrasion wounds, largely to test antimicrobial photodynamic therapy (aPDT).^37^ aPDT involves the combination of a non-toxic dye called a photosensitizer (PS) together with harmless visible light to excite the PS to produce reactive oxygen species that kill the microbial cells without harming the host tissue.^38^ The first report concerned excision-type dermal wounds on the mouse dorsal surface that were infected with bioluminescent *Escherichia coli* DH5α (Fig. 3).^36^ Because this particular strain of *E. coli* is non-invasive, the infection was self-limiting and multiple wounds could be constructed on a single mouse to allow the testing of a treatment such as aPDT with different wounds acting as appropriate controls. They showed that mouse excisional wounds infected by a virulent strain of bioluminescent *P. aeruginosa* could be successfully treated with aPDT, saving mice from death due to sepsis.^39^ Subsequent studies went on to study excision wounds infected with bioluminescent *Proteus mirabilis* treated with aPDT mediated by a cationic fullerene,^40^ and excisional wounds infected with *P. aeruginosa, P. mirabilis* and *S. aureus* that were treated by application of an antimicrobial chitosan acetate bandage.^40^

**Figure 4.**
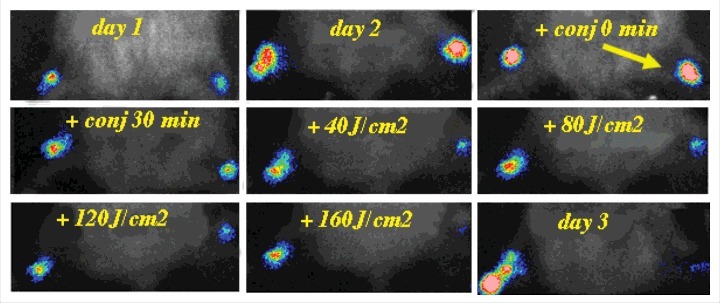
BLI of an immunosuppressed mouse model of deep soft tissue infection infected with *S. aureus* and treated with PDT. Figure adapted from data in.^30^

Two different models of infected skin abrasions were developed by the Hamblin lab. The first consisted of an overlapping series of needle scratches that could develop an infection by methicillin-resistant *S. aureus* (MRSA) (Fig. 4).^41^ In order for the infection to become established the mice need to be rendered temporarily neutropenic. This was accomplished by administering two successive IP injections of cyclophosphamide, the first of 100 mg/kg 4 days before wounding and the second of 150 mg/kg 1 day before wounding. The second model involved removal of a superficial layer of epidermis by scraping with a scalpel blade or by using “sandpaper”, that could be infected with *C. albicans*.^42^ See Bioluminescent reporter systems in fungi' section for a discussion about the genetic engineering necessary to produce bioluminescent *Candida* and other fungal species.

### Skin and soft tissue infection (SSTI) models

**Figure 5.**
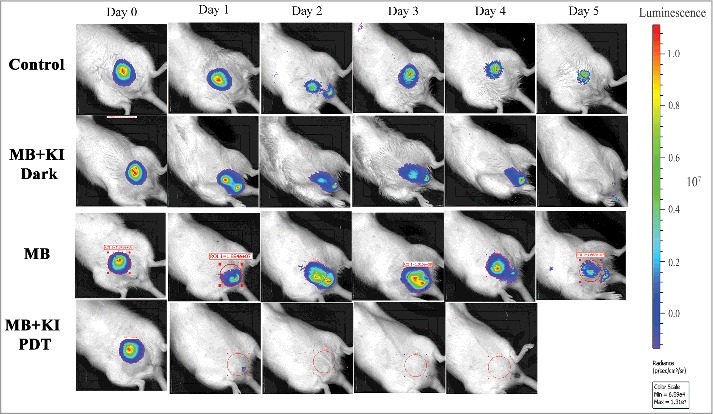
BLI of a female rat model of urinary tract infection with uropathogenic *E. coli* and treated with PDT. Unpublished data.

SSTIs are a rapidly progressing cause of morbidity and an uncommon, but significant cause of mortality, which may cause necrosis, abscesses and ulcers. In some cases, the causative organism is not identified. The emergence of multi-drug resistant organisms in SSTI has further placed a huge burden on health care management. Gad et al. devised a model of deep-tissue abscesses infected with bioluminescent *S. aureus* (Fig. 5).^30^ The mice needed to be rendered neutropenic with cyclophosphamide as described above. The model was used to test aPDT accomplished by injecting a solution of the photosensitizer into the infected area, followed by illumination with a surface spot of red laser light. A two-leg infection model was employed to allow the non-treated left leg to act as an internal control.

Recently, an anti-microbial nanofiber wound dressing including a nisin-eluting scaffold showed a significant reduction in *S. aureus* Xen36 as evidenced by BLI in a murine excision dermal infection model.^43^ In another study, *in-vivo* imaging technologies like BLI and 19F-MRI using perfluorocarbon were found effective for visualization of the effect of antibiotic therapy (vancomycin or linezolid) in a local *S. aureus* infection.^44^ The efficacy of different systemic and topical antibiotics against community-acquired MRSA (CA-MRSA) infected full-thickness dermal wounds was evaluated by BLI to monitor the bacterial burden in mice. Infection is the main cause of failure of implanted prosthetic biomaterials owing to peri- or early post-operative bacterial contamination. The progression of a biomaterial-associated infection (BAI) in real-time was demonstrated by Engelsman et al^44^ using surgical meshes with adherent *S. aureus* Xen29 in a soft tissue implant model in mice. Both bacterial growth and invasion into the surrounding tissue was monitored longitudinally by BLI. The study reported that the bioluminescence spread beyond the mesh area into surrounding tissues, presumably due to the “foreign body effect”. Recently, the same group compared the persistence of *S. aureus* Xen29 on and around both degradable and non-degradable surgical meshes that had been subcutaneously implanted in mice and monitored by longitudinal BLI. They showed that the use of biodegradable biomaterials yields major advantages (compared to non-biodegradable materials) with respect to the prevention of biofilm growth as well as allowing the host immune system to clear the bacteria.^45^ Local spread of *S. aureus* in a skin infection model in mice has been demonstrated non-invasively by using BLI. This study showed that the presence of coagulase enzymess that trigger fibrin formation together with staphylokinase that functions as a plasminogen activator, contributed to *S. aureus* skin infection by enhancing bacterial spread as a result of both fibrinolysis and proteolysis.^46^

### Burn infections

Burn injury is one of the most devastating types of damage that can compromise the defensive role of the skin. Burn wounds are highly susceptible to microbial infection leading to poor wound healing, development of systemic infection and even death. BLI has been widely used to study burn infection with a variety of pathogens and the treatment modalities. Burns in experimental animals that have been infected with different strains of bioluminescent bacteria such as *P. aeruginosa*,^47^ *Acinetobacter baumannii*^29,47,48^ and MRSA^49^ have been longitudinally monitored by BLI.

### Osteomyelitis infection model

Osteomyelitis is the infection of bone and sometimes bone marrow, typically arising after trauma that damages bone tissue, or can be caused by systemic spread of infectious microbes to bone tissue, or localized spread within the tissue that eventually reaches bone.^50^ Osteomyelitis is particularly dangerous due to the host response; as leukocytes enter the infected bone tissue region, they attempt to engulf bacteria and in the process release lytic enzymes that further break down the bone matrix.^51^ Osteomyelitis is often caused by *S. aureus* and *Streptococcus spp*. Bones with high vascularization and marrow content, including the femur, humerus, maxilla, tibia, and vertebra are most commonly the site of osteomyelitis infection.

In 2008, Li et al. designed a murine model of osteomyelitis by coating an orthopedic pin with *lux*ABCDE transformed *S. aureus* (Xen29) and monitored the osteolytic kinetics and the immune response. After implantation of the infected pin, osteolysis, occurrence of sequestrum (dead bone which separates from healthy bone), and biofilm formation were noted.^52^ BLI imaging was combined with *nuc* real-time quantitative PCR to monitor the bacterial growth. Both techniques revealed that 4 days post-implantation, the infection reached the greatest microbial burden which was then followed by biofilm growth at a lower metabolic rate. A similar technique was used to show that bone marrow could harbor localized listeriosis.^53^ Funao et al. created a BLI model of *S. aureus* osteomyelitis involving femur infection, which may be used to model chronic osteomyelitis that occurs in diabetic patients.^54^ They observed peak photonic emission from the same *S. aureus* Xen29 strain at 3 days post-infection, that remained high for 7 days.

BLI monitoring of osteomyelitis has been used to test potential anti-microbial techniques. Bisland et al. created a dual tibial *S. aureus* osteomyelitis model using rats and used this model to monitor the effect of aPDT.^55^ PDT was performed using the photosensitizer-precursor, 5-aminolevulinic acid (5-ALA), which leads to excessive endogenous production of protoporphyrin IX (or coproporphyrin in the case of *S. aureus*), that in turn acts as a photosensitizer. Intraperitoneal injection with 300 mg kg^−1^ 5-ALA was carried out and after 4 h the rat tibias were irradiated transcutaneously with 75 J cm^−2^ of 635 ± 10 nm laser light. One day post-treatment, bioluminescence was monitored. A decrease in bioluminescent signal (approximately 40%) was observed 24 h after treatment, although 48 h after treatment, the bioluminescent signal reduction was only about 20%. These statistically significant yet relatively poor reductions in viable cell counts may be explained by several issues. First and foremost, bone is not easily irradiated due to the scattering effect of the dense collagen and hydroxyapatite matrix. Moreover, the photosensitizer choice for PDT is not necessarily optimal: typically, cationic phenothiazinium dyes (such as methylene blue or toluidine blue O, etc.) work very well in the elimination of Gram-positive pathogens.^56^ 5-ALA was probably chosen seeing as it is already approved by the US Food and Drug Administration (FDA) for the PDT treatment of several neoplastic conditions.^57^ Despite these issues, the Bisland work is an excellent proof-of-concept paper for using BLI to monitor PDT for osteomyelitis.

Studies have been performed for the development of suitable *in-vivo* models of osteomyelitis (and several other bacterial infections) using the BLI technique to monitor therapeutic interventions in real-time. A range of these models is listed in Table 3.

**Table 3.** Other bacterial infections monitored by BLI.

Disease	Microorganism	Anatomical location	Model	Study/ Treatment	Refs
Osteomyelitis	*S. aureus* (Xen29)	Bone	Orthopedic pin pre-coated bacteria, implanted transcortically through tibial metaphysis	After implantation, osteolysis, sequestrum and biofilm occurred. 4 days post-implantation, the infection reached its peak, followed by biofilm development	^37^
Osteomyelitis	*Listeria monocytogenes*	Bone	A mouse model developed for *Listeria* bone infections	BLI revealed that bacteria grow in discrete foci and suggested that bone marrow could harbor localized listeriosis.	^38,53^
Osteomyelitis	*S. aureus*	Bone	A mouse model infected with bioluminescent *S. aureus* in the femur monitored by BLI	The model was found to be useful for elucidating the pathophysiology of both acute and chronic osteomyelitis (in case of diabetes) and to evaluate the effects of novel antibiotics or antibacterial implants	^39,54^
Osteomyelitis	*S. aureus*	Bone	Bacterial biofilm-coated K-wires were implanted into tibial medullary cavity of rats and monitored by BLI.	PDT using methylene blue (MB) or 5-aminolevulinic acid (ALA)	^55^
Osteomyelitis	*S. aureus*	Bone	An L-shaped, Kirschner-wire was transfixed into the L4 spinous process of 12-week-old C57BL/6 mice and inoculated with *S. aureus*	Vancomycin and rifampin combination therapy was superior to monotherapy	^212^
Gastrointestinal	*Salmonella typhimurium*	Intestines, mesenteric lymph nodes (MLN)	129sv Nramp1 (Slc11a1) mice	Bacteria persist within macrophages in the MLN but mice remain disease-free. An interferon-γ neutralizing antibody led to acute systemic infection	^59^
Gastrointestinal	*E. coli* (EPEC & EHEC)	Intestine (visible after injection of air)	C57BL/6 mice orally inoculated	EPEC persistence longer if mice anesthetized with ketamine/xylazine	^63^
Gastrointestinal	*Enterococcus facaelis*	Whole body imaging of larvae	*Galleria melonella* larvae; bacteria injected into hemocoel	Increased insect melanization, indicated that *E. faecalis* triggered a host innate immune response and activation of a prophenoloxidase cascade	^66^
Gastrointestinal	*Listeria monocytogenes*	intestines	Mice gavaged with bacteria	Probiotic bacteria *Lactobacillus plantarum* and *Enterococcus mundtii* could prevent colonization of the GI tract	^213^
Gastrointestinal	*Escherichia coli K1* A192PP-lux2	Tongue, esophagus, stomach, intestines, then brain, whole body	2 day old Wistar rat pups fed bacteria from dropper	*E. coli* K1 enters into the systemic circulation via esophagus	^214^
Urinary tract	*E. coli* UTI89	Bladder	Female Sprague-Dawley rats inoculated transurethrally	Enhanced susceptibility with neurogenic bladder caused by spinal cord injury	^70^
Mycobacterial tuberculosis	*Mycobacterium tuberculosis, M. smegmatis*	Lungs	Intranasal innoculation of CB-17 SCID mice	MTB transformed with a plasmid encoding red-shifted FLuc (FFlucRT) allowed monitoring of efficacy of isoniazid therapy	^76^
Mycobacterial Buruli ulcer	*M. ulcerans*	Footpad	Innoculation into footpad of female BALB/c mice	*M. ulcerans* transformed with plasmid encoding LuxCDABE allowed monitoring of efficacy of drug therapy	^22^
Endodontic	*P. aeruginosa, C. albicans*	Explanted teeth	Innoculation into root canals	Allows investigation of root canal geometry and aPDT	^80,82,83^
Bacterial pneumonia	*S. pneumoniae* (A66.1, a type 3 encapsulated strain)	Lungs	Female BALB/c mice inoculated intranasally	Adjunctive dexamethasone improved ampicillin outcomes in mice with severe pneumonia	^215^
Lung infection cystic fibrosis	*P. aeruginosa* TBCF10839 and D8A6 mutant	Lungs	C3H/HeN mice by intratracheal instillation	TBCF10839 strain caused 50% mortality while attenuated D8A6 allows monitoring of infection	^216^
Otitis media	*H. influenzae*	Nasopharynx, eustachian tubes, middle ear	Chinchillas inoculated intranasal and transbullar	Signal persisted for 10 days, but at later times did not correlate with CFUs from nasal lavage fluids suggesting formation of a biofilm	^95^
Otitis media	*S. pneumoniae* (Xen10)	Eustachian tubes, middle ear	Chinchillas inoculated into the epitympanic bullae	Antibiotics reduced duration of signal from 14 days to 2 days	^217^
Meningitis	*Neisseria meningitidis*	Whole body, brain, spinal cord	CD46 transgenic mice inoculated IV	Three patterns of disease (fatal, meningitis-like, or mild) were observed.	^99^
Meningitis	*S. pneumoniae* A 66.1 serotype 3	Brain, spinal cord	Bacteria inoculated into cisterna magna of immunocompetent hairless mice	Dexamethasone combined with daptomycin or vancomycin gave best results	^100^
Meningitis	*Cronobacter sakazakii*	Brain, liver, spleen, kidney, and gastrointestinal tract	Oral inoculation to Balb/c mice	Emerging food-transmitted pathogen (previously *Enterobacter sakazakii*)	^218^
Biofilm	*P. aeruginosa* (Xen29)*, S. aureus* (Xen5)	Flank region of mice	Insertion of a pre-colonized Teflon catheter segment (1 cm) under flank skin of BALB/c female mice	Infections persisted for >10 days and signal correlated well with CFU	^219^
Biofilm	*S. epidermidis* (Xen43)	Flank region of mice	Insertion of a pre-colonized Teflon catheter segment (1 cm) under flank skin of normal, SCID or nude mice	Nude mice were most susceptible, followed by SCID and Balb/c	^220^
Biofilm	*S. aureus* (MRSA and MSSA)	Flank region of mice	Implantation of a *S. aureus* precolonized Teflon catheter segment (1 cm)	Tedizolid was superior to linezolid or vancomycin for treatment	^221^
Biofilm	*S. aureus* Xen36 *S. epidermidis* Xen43	Dorsal region of SD male rats	Implantation of a precolonized section of venous access port catheter	Cyclophosphamide immunosuppression led to systemic infection. Antibiotic lock therapy could be tested.	^109^
	*P. aeruginosa Lm1, E. coli EAEC 55989*				
Biofilm endocarditis	*S. aureus* Xen29	heart	Indwelling polyethylene catheter in the left ventricle of rats to produce sterile vegetations followed by IV inoculation of bacteria	Vancomycin was superior to cefazolin, gentamycin was ineffective	^110^

### Gastrointestinal tract infection models

*Salmonella* enteric species such as *typhimurium, typhi*, and *enteriditis* are Gram-negative, facultative intracellular bacteria and cause a number of human infections wordwide.^58^ The use of BLI for longitudinal monitoring of bacterial infection was first demonstrated using *S. typhimurium* which had been genetically constructed to express *lux* *operon*.^17^ In this study, groups of mice were orally infected with three different strains of *Salmonella*, each expressing *lux genes* from a plasmid encoding *Lux operon*. The authors found that the course of infection could be either long-term chronic, or self-regulating, and the efficacy of antibiotic treatment could be monitored non-invasively in real-time.^17^

Monack et al^59^ performed an *in-vivo* study using BLI to monitor *S. typhimurium* chronic disease. Mice infected with *S. typhimurium* for 80 days exhibited higher bioluminescence signals, and immunohistochemical examination of the mesenteric lymph nodes showed that bacteria did not co-localize with neutrophils; but rather the bacteria were localized within different larger host cells that were surrounded by neutrophils. New-born and young children are highly susceptible to infection by *S. typhimurium*. BLI was used to study the effect of age on the susceptibility to this pathogen in BALB/c mice, by monitoring the progression of infection in different age groups: neonatal (1-wk-old), suckling (2-wk-old), juvenile (4-wk-old), and adult (6-wk-old). Mice were infected orally with various numbers of CFU of a bioluminescent *S. typhimurium* strain, and the infection was followed for 2 weeks. They showed that susceptibility to infection with *S. typhimurium* decreased with age.^60^ In 2007 the same group^61^ used BLI to analyze vaccine strains of *S. typhimurium* in a neonatal mouse model, and found that neonatal mice were not susceptible to infection even with high doses of the aroA-knockout mutant of *S. typhimurium*. In addition, the aroA–mutant survived for a prolonged time and stimulated both adaptive and protective immune responses, and therefore was considered a good candidate to be a vaccine strain for children.

Recently, Ozkaya et al.^62^ compared tissue bioluminescence with standard clinical scores as markers of Salmonella disease progression of BALB/c mice. Clinical scores comprised visual examination for motility, ruffled fur, hunched position, feeding, ataxia, tremors, and they were correlated with the bioluminescence images. The bioluminescence signal moved from the abdominal region (initial site) to distant tissue sites, demonstrating systemic infection. As the infection progressed the bioluminescence signal became stronger as well as more anatomically disseminated.

Rhee et al.^63^ developed a novel murine model to study diarrhea caused by infection with enteropathogenic *E. coli* (EPEC) and enterohemorrhagic *E. coli* (EHEC) using BLI and bioluminescent bacteria. EPEC and EHEC bacteria were transformed with a lux plasmid that includes constitutively expressed OmpC promoter. C57BL/6 mice were inoculated orally with bioluminescent EPEC or EHEC, and the bacteria in the intestines were detected using BLI in both *ex-vivo* and *in-vivo*. 3 days after infection, both strains were observed in the cecum and colon and there was no difference between bioluminescent non-bioluminescent EPEC strains. Although EPEC peaked on days 2–3, and was undetectable by day 7, when EPEC infected mice were anesthetized with xylazine/ketamine for imaging, the bioluminescence persisted strongly for up to 31 days. This surprising result was attributed to the possible anti-inflammatory effects of ketamine.^64,65^

La Rosa and coworkers investigated the pathogenesis of different *Enterococcus faecalis (E. faecalis)* strains.^66^ *E. faecalis* is generally considered to be part of the indigenous flora that inhabits the mammalian gastrointestinal tract (GIT), but has recently emerged as an important nocosomial pathogen producing hospital-acquired infections in the urinary tract, bloodstream, endocardial, and surgical sites. Cytolysin and gelatinase have been implicated as virulence factors in highly pathogenic strains. La Rosa used *E. faecalis* strains expressing the luxABCDE cassette under the control of either the P16S, cytolysin, or gelatinase promoters in an invertebrate infection model using *Galleria mellonella* caterpillars, and also in mice.^67^ Systemic infection of *G. mellonella* with bioluminescent *E. faecalis* MMH594 showed the activity of both the gelatinase and cytolysin promoters and the authors suggested that these virulence traits were host environment dependent. After pre-administration of oral antibiotics, efficient but strain dependent gut colonization was achieved. Bioluminescence signal obtained from the murine gut was found to be well correlated with the CFU counts.

### Urinary tract infection (UTI) models

UTI are particularly difficult to treat with antibiotics at the best of times, but now with the rise in antibiotic resistance, have become even more problematic.^68^ They are especially common in patients with spinal cord injury who need repeated catheterization.^69^ Patrick Seed's group^70^ has created a model of UTI using a uropathogenic *E. coli* (UPEC) strain originally derived from a clinical cystitis isolate (UTI89)^71^ that had been engineered with the luxCDABE operon. They used a model of female Sprague-Dawley rats inoculated in the bladder with 3.5 × 10(6) CFU. Rats with spinal cord injury (T10 complete transection) were much more susceptible to infection (3.5 × 10(3) CFU). In our laboratory we repeated this model of rat UTI monitored with BLI in order to test intravesical aPDT as a potential therapy for bacterial cystitis (see Fig. 5, unpublished data)

### Mycobacterial infection models

Due to the emergence of multidrug-resistant and extremely drug-resistant strains, the mortality caused by Mycobacterium tuberculosis infection has increased over time. The slow *in vitro* growth and highly infectious nature of *Mycobacterium* spp. present difficulties in models used in the laboratory for drug discovery, vaccines or treatment approaches against this highly virulent pathogen. To overcome these difficulties, the use of optical reporter systems has been considered.^72^ It has been demonstrated that *M. aurum* can act as a non-pathogenic, non-hazardous and predictive surrogate microorganism instead of *Mycobacterium tuberculosis *(*M. tuberculosis*) itself, allowing BLI to be used in anti-mycobacterial drug discovery.^73^ Anti-tuberculosis drug screening has been reported using bioluminescent *M. tuberculosis* reporter strains both in-vitro and inside macrophages,^74,75^ and also in *in-vivo* mouse models.^76^

BLI has been used to monitor animal models of pulmonary tuberculosis. Using integrating vectors, the *in-vivo* detection of bioluminescence in the lungs of mice infected with either Fluc-expressing *M. smegmatis* or *M. tuberculosis*, or *lux-*expressing *M. smegmatis* was assessed. However, the group reported the need to use a very high bacterial inoculum in comparison with the usual levels inoculated in mouse studies of infection by *M. tuberculosis*. The obtained signal was stronger when using the intraperitoneal rather than the intranasal route to administer the luciferin.^77^

*M. ulcerans* is the causative agent for an ulcerative skin disease so called Buruli ulcer. Using a mouse footpad model, Zhang et al. investigated the use of recombinant *M. ulcerans* strain expressing the *lux*AB gene from Vibrio harveyi for *in-vivo* real-time BLI monitoring of potential anti-mycobacterial treatments.^22^ While the recombinant *M.ulcerans* strain and the wild-type strain were both found to be similar in terms of virulence and drug susceptibility and BLI shortened the time needed for the assessment of new drugs, the proposed system still had limitations such as the requirement of repeated injections of the exogenous substrate needed for the luciferase reaction and the substrate's poor diffusion, which possibly reduced sensitivity. The same group also demonstrated a potential high-throughput method for rapid, serial, real-time *in-vitro*, and *in-vivo* assessment of anti-tuberculosis drug and vaccine efficacy, via employing autoluminescent *M. tuberculosis* reporter strains expressing *lux*CDABE.^78^ While minimum of 4 weeks is generally required to distinguish active from inactive tuberculosis drugs, BLI was able to reduce this process to less than 5 days. Moreover, vaccine efficacy could be demonstrated only within 3 weeks. Nevertheless, the authors mentioned that the integrated *lux*CDABE was not fully stable and non-luminescent revertants existed even upon application of an exogenous substrate. An additional limitation of this method was the need for high bacterial burden required for detection.

### Endodontic infection models

Endodontic infections are polymicrobial, and are made up of predominantly anaerobic bacteria with some facultative bacteria. Endodontic therapy is designed to eradicate the pathogenic bacteria from the root canal system during chemical and mechanical endodontic treatment. The bacterial infection has a significant role in dental pulp necrosis and periapical lesion development.^79^ Studies using *in-vitro* and *in-vivo* models commonly employed microbiological culture methods, which posses several limitations such as inability to get complete bacterial density from the sample of root canal, and the need to monitor sequential procedures using CFU counting.^80^

Sedgley et al. used a bioluminescent reporter strain *Pseudomonas fluorescens* 5RL containing a lux CDABE plasmid to study the mechanical efficacy of irrigation to reduce bacterial load in the root canal and whether the depth of placement of the irrigation needle made a difference.^81^ In another study Sedgley et al. used *in-vitro* live BLI with the bioluminescent reporter strain, *P. fluorescens* 5RL to quantify root-canal bacteria after sequential treatment.^82^ The same *P. fluorescens* strain was used to determine whether the root canal curvature made a difference on the efficacy of root canal irrigation *in-vitro* using BLI.

**Figure 6.**
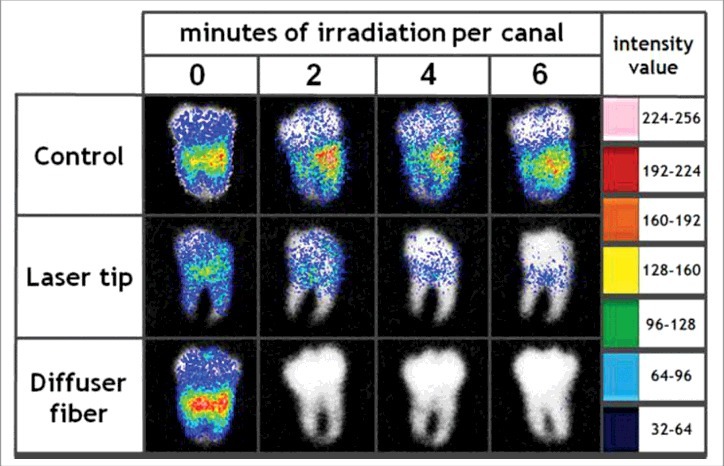
BLI of explanted intact third molar human teeth with *C. albicans* endodontic infection and treated with PDT. Figure adapted from data in^83^

Researchers have studied a combination treatment applying PDT togther with mechanical removal for effective treatment of endodontic infection. Garcez et al. used bioluminescent *P. aeruginosa* (XEN5) due to its high bioluminescence signal and its ability to form biofilms in the root canal. Antimicrobial-PDT combined with endodontic therapy improved the ability to eliminate bacterial biofilms. Endodontic therapy decreased bioluminescence signal by 90%, PDT reduced it by 95% and combination therapy resulted in more than 98% reduction. Fig. 6 shows the representative bioluminescence images captured from teeth infected with 3-day *P. aeruginosa* biofilms.^31^ Sabino et al. used an *in vitro* model with bioluminescent *C. albicans* biofilms formed inside curved root canals to investigate different light delivery methods for antimicrobial PDT (using methylene blue and red laser light). They found that light distribution in the root canal was markedly dependent on the light delivery system, with an optical diffusing fiber, giving 100 times better reduction in microbial burden than a flat tip fiber.^83^

### Lung infection models

BLI has been used by many researchers for the study of lung infections. Given the limitations of BLI when applied to organs that are far from the surface, there are a number of studies addressing the optimal conditions to take advantage of this technique in the context of this organ system. The depth and opacity of the tissues complicates the signal acquisition from the lungs^84^ so that the photon counts obtained *in-vivo* from the lungs of mice are 100- to 1000-fold lower compared to the *ex-vivo* analysis. Likewise, *in-vivo* analysis of the lungs of BALB/c mice gives higher bioluminescence signals than those from C57BL/6 mice. This is in agreement with the 10-fold reduction of light transmission due to the dark fur and pigmented skin of C57BL/6 mice in comparison with hairless mice or albino mice.^21^ Thus, obtaining relevant results about the infectious process in the lungs can be highly dependent on the chosen model. Bioluminescent strains of *S. Pneumoniae* allowed the modeling of bacterial pneumonia in mice. A study conducted using a pneumococcal lung infection model demonstrated the effectiveness of integrating the *lux* genes into the chromosome of Gram-positive bacteria using the Tn*4001luxABCDE*Km^r^ transposon cassette. This achievement improved the *in-vivo* monitoring of viable bacterial cells compared with the previously generated *S. pneumoniae* strain carrying a modified version of the operon in a plasmid, that tended to lose plasmid expression in the absence of antibiotic selection.^13^ The aforementioned lux transposon cassette allowed modeling of the course of pneumococcal infection in mice infected with specific strains of *S. pneumoniae*.^85^ Henken et al. used BLI to compare invasive and non-invasive bacterial infections in the lungs of mice. They infected two different mouse strains with either the less virulent serotype-19 *S. pneumoniae* or the invasive serotype-2 *S. pneumoniae*, both expressing the *luxABCDE* operon. The analysis revealed the highest correlation between *in-vivo* bioluminescent signal and CFU counts were observed on the third day post-infection with serotype-2 *S. pneumoniae* delivery via the intratracheal route.^86^ *S. pneumoniae* is also considered to be the major pathogenic agent involved in the development of lung complications after influenza virus infection. Since this problem has been reported in both adults and in children, researchers have carried out the sequential imaging of this infection using the above mentioned bioluminescent pneumococcal serotypes in both infant and adult mice.^85^ Short et al. developed a mouse model to investigate the mechanisms involved in the synergistic relationship between *S. pneumoniae* and the influenza A virus. Utilization of BLI enabled monitoring of infection progression as well as the kinetics of pneumococcal transmission.^21^ Lastly, the use of a bioluminescent *P. aeruginosa* bacterial strain highlighted the ability of bacteriophages to combat and prevent bacterial lung infections.^87^

### Otitis media infection models

Middle ear or otitis media infections (OMI) are frequently observed in children, and can be caused by *S. pneumoniae*^88^ *P. aeruginosa*,^89^ non-typeable *Haemophilus influenza*,^90^ or *Moraxella catarrhalis*.^91^ OMI is seen in 70% of the children making it one of the leading pediatric diagnoses. Increased insight into the biofilm forming bacteria elucidated the pathophysiology of OMI.^92^ Various animal models have been utilized such as infant/ adult mice, rats, infant rhesus monkey, gerbils, however, adolescent/ adult chinchillas are still preferred for acute OMI, since the model was first developed in 1975 at the University of Minnesota.^93^

Current mouse models have some limitations as the infection is initiated through an invasive procedure while larger animals like chinchillas and ferrets have natural routes of infection. Chaney et al. reported induction of a non-invasive middle-ear biofilm infection in rats through repeated bacterial inoculation combined with pressure changes in the ear.^94^ Novotny et al. transformed a non-typeable *H. influenzae* clinical isolate with a plasmid containing the luxCDABE operon. Authors studied the ability to detect bioluminescence and infection progression in eustachin tubes and middle ears of chinchillas via inoculating through intranasal transbullar routes.^95^ *S. Pneumoniae* OMI can occur as a secondary bacterial infection following an initial influenza virus infection. Peltola et al.^96^ demonstrated that, when challenged with a bioluminescent *S. Pneumoniae*, ninety percent of ferrets infected with the H3N2 virus developed OMI while this rate was only ten percent for the ferrets that were infected with H1N1 or influenza B virus. Ninety percent of ferrets infected with the H3N2 virus developed OMI while only 10 percent of the ferrets developed OMI that were infected either with H1N1 or influenza B virus. Successful results achieved by this model suggest that it can be further utilized to study pathophysiology of otitis media and sinusitis infections especially those that stem from viral-bacterial synergism.

### Meningitis infection models

Meningitis is an inflammation of the membranes covering brain and spinal cord, which are called as meninges. Various microorganisms such as virus, bacteria, fungi and parasites can cause meningitis, and when not treated it is often times life-threatening.^97^

Sjölinde et al. investigated how the meningococci bacteria localized in CD46 transgenic mice using *in-vivo* BLI to observe the disease dynamics during meningococcal infection.^98^ In another study BLI was used in a mouse model of *Neisseria meningitides* infection, to test treatments that could improve outcomes in patients suffering from meningitis.^99^ Mook-Kanamori et al. tested the antibiotic daptomycin (a lipopeptide) in a murine model of pneumococcal meningitis caused by *S. pneumoniae*. Mice were inoculated intracisternally (into a brain cavity) with serotype 3 *S. pneumoniae* possessing an integrated lux operon. Caspase-3 staining was used to detect apoptosis in brain histopathological slices, and they also measured bioluminescence and numbers of bacterial CFUs in the cerebrospinal fluid (CSF).^100^ Different light emission spectra and substrates required for lux and Fuc, enabled the separate monitoring of two different bioluminescence reporters which in turn made it possible to evaluate disease progression and the therapy response.^101^ Based on the different spectral light emission and substrate requirements for lux and Fuc, the group was able to separately monitor the two bioluminescence reporters using a highly sensitive BLI system and thereby evaluate the disease progression as well as the response to therapy.^101^

### Biofilm infection models

Biofilm contains complex group of adharent microorganisms within a polymeric matrix which is made of exopolysaccharides (EPS) produced by the microbial cells.^102,103^ Pathogenesis of several infections such as gingivitis, caries, periodontitis, middle-ear infections, urinary tract and catheter infections involve biofilms.^104^ Several studies have described *in-vivo* models that allow a real-time monitoring of the biofilm infections using BLI. Implanted devices or internal prostheses are highly prone to infection, and BLI can be used to study these infections that have points of high clinical relevance. It enables to investigate the role of immune system in biofilm infections and also facilitates monitoring of response to treatments.

Lönn-Stensrud et al.^105^ showed the action of different furanones could decrease biofilm formation of the bioluminescent S*taphylococcus epidermidis* (*S. epidermidis*), without anti-microbial, irritative or genotoxic effects. They concluded that two candidate furanones (out of the 11 screened) could inhibit biofilm formation by interfering with quorum sensing, and thus could be promising agents for preventing surface colonization by *S. epidermidis*. Recently, Pribaz et al^106^ developed a model of a chronic *S. aureus* biofilm infection which commonly arises post-arthroplasty (knee joint replacement). A stainless steel implant placed into the knee joints of mice was inoculated with one of the 4 different strains of *S. aureus* and infection progression was monitored for 42 days via BLI. One strain had the bioluminescent construct (luxABSCDE) in an antibiotic selection plasmid (ALC2906), the other two strains had lux gene integrated into the bacterial chromosome (Xen29 and Xen40), while the fourth strain had the lux genes in a stable plasmid (Xen36). The authors concluded that in all strains biofilm formation was comparable; Xen29, Xen40 and especially Xen36 (which had the stable bioluminescent construct) were useful for long-term *in-vivo* monitoring of chronic post-arthroplasty infections and the effectiveness of potential therapeutic interventions. Engelsman et al.^107^ studied a model using surgical meshes cultured with pre-adherent bioluminescent *S.aureus* Xen29, which were subsequently implanted in mice. Bacterial growth as well as invasion into the surrounding tissue was longitudinally monitored via BLI. Bioluminescence values obtained prior to sacrifice were correlated with the number of organisms isolated from the removed implants. Based on the results, the authors concluded that BLI is a potential alternative to *in vitro* studies, as it enables long-term *in vivo* evaluation of anti-microbial coatings without the need to obtain explanted meshes and entails a major factor lacking *in vitro* studies – the host immune system.

Niska et al.^108^ investigated the effectiveness of several antibiotics (vancomycin, daptomycin and tigecycline) in prophylaxis of surgical implant infections. In a mouse model of biofilm-infection, the knee joints of mice were fitted with a surgically placed medical-gradee metal implant, and bioluminescent strains of MRSA (USA300 LAC:*lux*) or methicillin-sensitive *S. aureus* (MSSA) (Xen36) were then inoculated into the joint cavity. Both bioluminescent strains enabled evaluation of prophylactic therapy efficacy at different doses.

Chauhan et al.^109^ studied infections that occur on a pediatric implantable venous access port (PIVAP). They used an *in-vivo* bioluminescence model of chronic bacterial biofilm infections in a surgically placed PIVAP in both immunocompetent and immunosuppressed rats. They showed that 70% of immunocompetent rats were able to prevent the infection from becoming established and clear the bacteria from the bloodstream, while none of the immunosuppressed rats survived the infection. This model is expected to allow assessment of anti-biofilm and anti-thrombosis therapeutic interventions, as well as the optimization of long-term management of access ports.

Xiong et al^110^ studied a rat model of infective endocarditis (IE) in the aortic valve caused by a bioluminescent biofilm-producing *S. aureus* strain that was vancomycin and cefazolin susceptible but gentamicin resistant. Persistent and increasing bioluminescence signals were obtained from the untreated animals. Three days of vancomycin therapy led to significant reductions in both cardiac bioluminescence signals and the numbers of CFU in the cardiac vegetations. Cefazolin was less effective while gentamycin had no effect. However, 3 days after discontinuation of vancomycin therapy, the cardiac BLI and CFU recurred indicating that the IE had relapsed.

## BLI monitoring of animal models of infections induced by pathogenic fungi

Limitations in the current diagnostic methods for fungal infections, as well as the frequent development of resistance to antifungal drugs has led to an increased search for new therapeutics. BLI enables to understand and monitor the fungal infection processes, for drug discovery. The life cycle of most strains of *C. albicans* involves two developmental programs, that involve differential gene expression; bud-hypha transition^111^ and high-frequency phenotypic switching.^112^ In order to understand the regulation of differentially expressed genes, it is necessary to functionally characterize the promoters of genes that are expressed in a phase-specific manner and a bioluminescent reporter system can facilitate this process.^113^ Several methods have been developed for monitoring *C. albicans, Aspergillus spp* and *Neurospora crassa* infections, some of which are shown in Table 4.^114^

**Table 4.** Fungal infections monitored by BLI.

Disease	Microorganism	Animal host	Anatomical location	Type of luciferase	Model/ Treatment	Refs
Candidiasis	*C. albicans*	CD1 female mice immunosuppressed with cyclophosphamide	Superficial and subcutaneous	PGA59-GLuc fusion at cell surface, or ACT1 and HWP1 promoters	Some evidence for systemic infection in kidneys	^33^
Candidiasis	*C. albicans* B311 or ATCC 90234	Female Balb/c mice inoculated IV or IP Female mice given weekly SC injections of estradiol	Systemic (kidneys) or vulvo-vaginal	pGTV-ENO plasmid with codon-optimized FLuc under constitutive promoter	In both models fungal infection could be detected for over 30 days. Miconazole cleared vulvo-vaginal infection	^132^
Candidiasis	*C. albicans*	Oral inoculation of C57BL/5 mice treated with SC cortisone acetate every 2 days	Oropharyngeal	GLuc	Infection spread to esophagus and stomach	^25^
Candidiasis	*C. albicans*	*Galleria melonella* larvae	Whole body	GLuc	Allowed monitoring of fluconazole therapy	^222^
Biofilm infection	*C. albicans*	Female Balb/c mice with dexamethasone in drinking water	Implantation of pre-colonized catheter segments under dorsal skin of mice	GLuc (constitutively active ACT1 or HWPg hyphal promoter)	Strain with hyphal-specific FLuc allowed visualization of morphological transition	^134^
Aspergillosis	*A. fumigatus*	Intranasal inoculation of male Balb/c mice immunosuppressed with cyclophosphamide	Lungs	Codon-optimized FLuc	Liposomal amphotericin B gave best results	^128^
Aspergillosis	*Aspergillus terreus*	Balb/C mice immunosuppressed by cyclophosphamide or CD-1 mice with cortisone acetate IP	Lungs	Codon-optimized FLuc under gpdA promoter	Cyclophosphamide infection worse than corticosteroid	^223^

The two principal luciferase systems used in fungi are Fluc from *Photinus pyralis* and Gluc from *Gaussia princeps*. The presence of O_2_ and exogenous luciferase substrates; D-luciferin and coelenterazine (depending on the source of luciferase) are essential for the light-producing reactions. Their deficiency and/or their nonhomogenous distribution are considered to cause obstacles in BLI of disseminated candidiasis. Possible prevention of luciferin uptake by the less permeable cell wall in *C. albicans* hyphae, auto-oxidation and/or rapid clearance of substrates from the blood^115,116^ as well as light absorption of hemoglobin and tissue should also be taken into account while monitoring systemic candidiasis via BLI. Light emission intensity decreases approximately by a factor of 10 for each cm of tissue depth.^17^ Thus, FLuc with emission in the red to infrared (>600 nm) might be preferable due to diminished light absorption by tissue and hemoglobin at these wavelengths.^117^ Lastly, FLuc oxidizes its substrate in an ATP-dependent manner generating oxyluciferin, AMP, CO_2_, and light.^118^ Therefore Block suggested that, apart from the cell wall structure, and the number of peroxisomes (where FLuc is localized), the ATP content may also be different in hyphae, which in turn would reduce substrate availability for the luciferase reaction.^119^

### Bioluminescent reporter systems in fungi

The first enzymatically active FLuc was produced in *Saccharomyces cerevisiae* (*S. Cerevisiae*) in 1988.^120^ However, the promoter used in this study led to a low level of expression. In an attempt to increase the level of expression, stronger promoters were utilized and the assay conditions were optimized.^121^ Nevertheless, the sensitivity was still too low. It was assumed that peroxisomal localization of native FLuc that was controlled by the C-terminal SKL sequence, might have limited the access to the exogenously administered enzyme substrate (luciferin) resulting in low levels of light emission.^119,122^ Indeed when Leskinen and colleagues removed the peroxisomal targeting codons, high levels of light emission were obtained.^122^ Moreover, cells with modified luciferase happened to grow at a much faster rate compared to those with the wild type luciferase.^122^

Similar to *S. cerevisiae*, initial methods to use FLuc for BLI of *C. albicans* also had several limitations. First of all, *C. albicans* has a different codon strategy, such that tRNA carries a CAG anticodon, to encode codon CUG as serine instead of leucine.^123^ On the other hand, FLuc contains 9 in-frame CUG motifs within its open reading frame.^124^ This phenomenon created a dysfunctional or unstable FLuc gene product causing low bioluminescence intensity. In order to overcome this obstacle, a bioluminescent *C. albicans* strain was developed by replacing CUG codons with UUG to enable functional expression.^125^ As a second alternative, FLuc was replaced with Renilla luciferase, as luciferase gene from *Renilla reniformis* does not contain CUGs.^113^ One of the major challenges faced in BLI of systemic candidiasis was the potential hampered diffusion of luciferin during the yeast-to-hyphae transition – a major virulence factor in this species.^126^ This limitation was tried to be eliminated via developing a novel reporter gene, GLuc59 which was constructed by fusion of a naturally secreted synthetic *G. princeps* luciferase gene with the *C. albicans PGA-59* gene that codes for a glycosyl-phosphatidyl-inositol-linked cell wall protein.^33^ Although the cell-wall-bound GLuc59 system was hundreds of fold more sensitive than the Renilla luciferase system and GLuc59 expression could also be detected during the hyphal development, no satisfactory results were obtained in monitoring progression of systemic infections.^33^ As discussed by Brock, this may be attributed to the limited distribution of GLuc59 substrate coelenterazine after intraperitoneal injection and to the sub-optimal emission wavelength of 480 nm that is probably absorbed well by hemoglobin.^119^

Bioluminescent reporters have also been constructed for studying infections that involve filamentous fungi.^127,128,129^ Brock et al. tested a new system in which the FLuc was codon optimized for mammalian cell expression, peroxisomal-targeting sequence was removed and the promoter region of the glyceraldehyde-3-phosphate dehydrogenase gene (*GpdA*) was used^119,127^ Glyceraldehyde-3-phosphate dehydrogenase plays a role in glycolysis and gluconeogenesis by reversibly catalyzing the oxidation and phosphorylation of glyceraldehyde-3-phosphate. Based on these facts, GpdA was assumed to be necessary for the metabolism of *A. fumigatus*.^127^ In contrast to the previous assumption, the fact that sufficient bioluminescence signal was obtained from filamentous cells indicated that the cell wall structure of the hyphae may not necessarily alter luciferin availability within the intracellular compartment. Nevertheless, in case of invasive bronchopulmondary Aspergillosis, starting from 24 h post-infection, despite the high fungal load, the bioluminescence signal intensity decreased significantly.^127^ Dissolved O_2_ is essentially required by all luciferases, thus the decline in bioluminescence intensity was mainly attributed to the hypoxic environment generated by the inflammatory process.^126^ Subsequently, Donat and colleagues developed an *A*. *fumigatus* strain which expressed a cell-surface exposed GLuc.^129^ This method allowed longitudinal monitoring of cutaneous Aspergillosis, however due to the low sensitivity, BLI monitoring of invasive pulmonary Aspergillosis was again not possible. Moreover homogenous distribution of the substrate, coelenterazine was still difficult to achieve. These limitations were similar to the ones observed with *C. albicans* expressing GLuc59.^130^

In order to investigate light induced activity and circadian activity in the non-pathogenic fungus *Neurospora crassa* using BLI, a fully codon-optimized FLuc gene was constructed, and a strong bioluminescent signal was obtained when fungal transformants were grown on media supplemented with luciferin.^131^

### BLI of candida albicans infections

In a vulvo-vaginal infection model of mice, BLI system enabled visualization of the *C. albicans* within the vaginal lumen via direct application of luciferin to the area.^132^ High correlation between light emission and numbers of CFU was achieved. Moreover, when a topical anti-fungal drug, miconazole was applied to the infected area, clearance of the infection could be validated by BLI. Despite these satisfactory results, when BLI was used in mouse models of systemic candidiasis, bioluminescence intensity was too low.^132^

Enjalbert et al.^33^ suggested the use of cell-wall bound GLuc system (GLuc59) for monitoring *C. albicans* infections. As earlier discussed, through construction of a luciferase, exposed at the cell surface, easy access of substrate to *C. albicans* was assured whether it was in the yeast or hyphal form. Consistent with these assumptions, no significant differences were observed in BLI between yeast cells and hyphae. Following application of the substrate, coelenterazine to the infected region, progression of cutaneous, subcutaneous, and vaginal infections could easily be monitored by BLI, and light intensities correlated with the numbers of CFU.

The efficacy of a conjugate vaccine against β-glucan that had been formulated with the human-compatible MF59 adjuvant, was evaluated in a murine vaginal candidiasis model.^133^ Extent, duration as well as level of protection from vaginal infection were monitored using GLuc59-expressing *C. albicans* strains. Based on the results, it was concluded that BLI was a more reliable method for assessment of vaginal infections than the CFU assay performed by sampling the vaginal cavity.^133^ This conclusion probably stemmed from fact that GLuc59 luciferase enabled more efficient detection of hyphal cells that did not easily form CFU.

Jacobsen and collegues were able to image systemic candidiasis by constructing a codon-optimized FLuc.^23^ To further enhance the bioluminescence signaling, the peroxisomal targeting sequence was removed. BLI of infected mice kidneys as well as the gall bladder provided valuable insights about both the disseminated infection process and also enabled to identify of sites of persistence. Surprisingly, subsequent to succsesful fluconazole and caspofungin treatments, viable *C. albicans* cells persisted in the gall bladder.^23^ The presence of *C. albicans* in the feces further indicated that gall bladder acts as a reservoir for colonization after therapy.

There were reports from the laboratory of Vecchiarelli looking at a mouse model of oropharyngeal candidiasis monitored by BLI.^25^ Mice were rendered susceptible by injection with cortisone acetate, and then a swab saturated with gLUC59-expessing strain of *C. albicans* was applied sublingually. They went on to show^24^ that corticosteroid-treated IL17a(−/−) mice developed invasive candidiasis following oropharyngeal infection, whereas wild-type mice did not. IL17a(−/−) mice showed significant infiltration of the fungal cells in the stomach. Increased permeability and mucosal ulcerations of the intestinal barrier favored *C. albicans* dissemination in the kidneys and liver. Neutrophils from IL17a(−/−) mice were as capable of phagocytosing the *C. albicans* cells as those of wild-type mice, but their candidacidal ability was less.

Fungal biofilms are highly resistant to most antifungal drugs therefore they are difficult to treat in clinical settings. Van Dijck and collegues for the first time used BLI as a modality to study *C. albicans* biofilm infections *in vitro* and *in vivo*.^134^ This method enabled to monitor both the time-course of biofilm formation as well as the changes in cell morphology during the process. By using a bioluminescent BCR1 deletion strain, the group was able to demonstrate the important role of BCR1 gene in substrate adhesion and biofilm formation.^134^ Subsequently, the group also introduced a new method for BLI of *C. albicans* biofilm formation on subcutaneously implanted catheters in mice and extracellularly located GLuc was used for this purpose.^135^

### BLI for aspergillus fumigatus infections

*A. fumigatus* is the major cause of invasive aspergillosis, a fungal disease that can occur in immunocompromised patients, and limited number of drugs are currently available for treatment. BLI was suggested as a potential modality for use in the development of novel anti-fungal agents and for providing new insights into the establishment and manifestation of the infection. In order to achieve this goal, Brock and collegues constructed a bioluminescent *A*. *fumigatus* strain by fusing the glyceraldehyde-3-phosphate dehydrogenase gene from *A. fumigatus* with the FLuc gene.^127^ The results were initially promising, in that light emission correlated with the number of conidia (non-motile spores) *in vitro*. In the same study, deep tissue infection could be also monitored by BLI but with some limitations. Corticosteroid-treated immunosuppressed mice were intranasally infected with *A. fumigatus strain* C3 and mice developed invasive aspergillosis.^127^ In order to monitor the infection using BLI, D-luciferin was injected intraperitoneally. The bioluminescence signal was only detected in lungs indicating that invasive aspergillosis was confined to the lower respiratory tract.^127^ However only early stages of pulmonary infection could be monitored. Possible reasons for failure to image late stages of pulmonary invasive aspergillosis have been previously discussed.

Donat et al. used an alternative method via using a bioluminescent *A. fumigatus* strain which expresses a cell surface-exposed GLuc.^129^ Although highly sensitive in longitudinal monitoring of cutaneous aspergillosis, this method also failed to reliably detect pulmonary aspergillosis.

## BLI monitoring of infections caused by eukaryotic parasites

Recently, various studies have demonstrated that BLI can also be used to study parasitic infections in live mammalian hosts. The ability to monitor specific stages of the parasite life cycle *in-vivo* is an important advancement for studying its pathogenesis (see Table 5 for some examples).

**Table 5.** Eukaryotic parasitic^175^ infections monitored by BLI.

Disease	Microorganism	Animal host	Anatomical location	Type of luciferase	Model/ Treatment	Refs
Malaria	*Plasmodium berghei* schizonts (pathogenic to rodents)	Swiss and C57BL/6 mice inoculated IV	Lungs, adipose tissue, spleen, whole body	FLuc under schizont-specific promoter ama1	Sequestration patterns of the schizont stage can be analyzed within 1–2 d after infection	^136^
Malaria	*P. berghei* sporozoites	Mice, C57BL/6 albino, C3H/HeNCrL, C57BL/6 WT, BALB/c, ICR/CD-1	Liver	FLuc	RLU values were in the following order: C57BL/6 albino, > C3H/HeNCrL, > C57BL/6 WT, > BALB/c, > ICR/CD-1 CFU highest in C57BL/6 WT suggesting the black skin blocked light	^141^
Trypanosomiasis Chagas disease	*Trypanosoma cruzi*	SCID mice inoculated IP	GI tract (colon & stomach)	red-shifted FLuc *PpyRE9h*	Mice developed myocarditis despite no parasites in the heart. Monitored treatment with benznidazole.	^224^
African trypanosomiasis Sleeping sickness	*T. vivax*	Swiss mice inoculated IP or SC	Spleen, lungs, liver, brain	FLuc (TvLrDNA-luc)	More rapid progression after IP inoculation, brain involvement at later stage	^149^
African trypanosomiasis Sleeping sickness	*T. brucei gambiense* strain 1135	Male & female BALB/c mice inoculated IP	Reproductive organs (ovaries; uterus; testes; seminal vesicles) brain, spinal cord, spleen	RLuc	Transmission may occur horizontally (sexual contact) and vertically	^225^
Leishmaniasis	*Leishmania major*	C57BL/6 mice inoculated in ear pinna dermis	Ear pinna	FLuc	Depending on inoculum, immune response can produce clinically silent niche with a small L. major population	^226,227^
Visceral Leishmaniasis	*L. infantum chagasi*	Male golden hamsters inoculated IP	Abdominal and pelvic organs	FLuc	Treatment with Glucantamine (Sb^V^) or miltefosine was monitored	^146^
Leishmaniasis	*L. amazonensis*	Female Balb/c mice inoculated in footpad or tail base	Footpad or tail base	FLuc	Treatment with amphotericin B was monitored	^146^
Toxoplasmosis	*Toxoplasma gondii* Virulent S23 and non-virulent S22	Female Balb/c mice inoculated IP	Visceral organs, lungs, brain	FLuc	Chronically infected mice could be reactivated with dexamethasone	^159^
Toxoplasmosis	*T. gondii* non-virulent CTGluc and virulent RH-LDMluc	Male Balb/c and IFNγR−/−mice inoculated IP	Visceral organs, brain	FLuc	Virulent strain and lack of IFNγ potentiated infection. Administration of hydrocortisone to asymptomatic mice after day 10 led to recrudescence in brain	^156^

### Malaria infection models

For the first time, Franke-Fayard et al.^136^ described a protocol for real-time *in vivo* BLI of blood stages of malaria parasites in mice. For this purpose, a mutant parasite was engineered by cloning the fusion gene GFP–luciferase under the control of the ama1 gene promoter of *Plasmodium berghei*. The localization of the schizont stage of *P. berghei* in live mice or in dissected organs could be quantitatively analyzed by BLI within a period of 24–48 h after infection.^136,137^ *In-vitro* and *in-vivo* drug activity luminescence assays (ITDL, IVDL) were reported for drug screening against blood stages of *P. berghei*. For the ITDL assay, luciferase activity of transgenic parasites with and without drugs was measured in order to quantify *in-vitro* transformation of sporozoites into mature schizonts. The IVDL assay was based on measuring luciferase activity of circulating parasites in samples of blood from the tail of mice which had been treated with candidate anti-malarial drugs.^138^

The transgenic *P. berghei* parasite (PbGFP-Luc_con_) that expressed luciferase was used to evaluate immunity against malaria. The authors concluded PbGFP-Luc_con_ parasites could be useful for studying prophylaxis against malaria and investigating the biological and immunological principles underlying protection.^139^ A transgenic *P. yoelli* strain was generated that expressed a luciferase reporter at all stages of the parasite life cycle.^140^ In-vivo BLI of these parasites made possible quantitative analysis of *P. yoelii* burden in the liver, and the parasite development could be compared with alternative assays using quantitative RT-PCR analysis of liver samples. Finally, the authors concluded that BLI was a rapid, simple and non-invasive method for monitoring pre-erythrocytic malaria infection that is useful for evaluation and screening the effects of anti-malarial drugs *in vivo* and in real-time. Recently Li et al. used BLI to compare the susceptibility of different mouse strains to liver infection using *P. berghei* sporozoites expressing Fluc.^141^ After injection of 10,000 *P. berghei* sporozoites, the relative light units (RLU) values were in the following order: C57BL/6 albino, > C3H/HeNCrL, > C57BL/6 WT, > BALB/c, > ICR/CD-1 for different mouse strains. However culture from mouse livers showed highest numbers in black C57BL/6 WT suggesting the black skin significantly reduced bioluminescence measurement.

### Leishmania infection models

Leishmania species, a protozoan parasite of the family *Trypanosomatidae*, causes different human diseases that range from benign cutaneous leishmaniasis to fatal visceral leishmaniasis. BLI using transgenic bioluminescent Leishmania cells can be used to investigate parasite virulence factors, elucidate immune regulatory mechanisms and can be used in the development of potentially new anti-leishmanial drugs.^142,143^ Transgenic luciferase-expressing Leishmania parasites introduced into small animal models either intradermally or intravenously, allow longitudinal monitoring of the parasitic load. Lang et al.^144^ used bioluminescent reporter Leishmania cells to monitor infection and response to therapy during high-throughput screening of drugs in in-vitro, in excised organs from infected mice, and in living mice. BLI with luciferase-expressing Leishmania and RT-PCR were combined to study the *L. major* or *L. donovani* intracellular amastigote burden and tissue transcript fluctuations to provide further insights on the complex interaction between Leishmania parasites and the mammalian host defense.^145^ Bioluminescence generated by recombinant *L. amazonensis* promastigotes and intracellular amastigotes has been shown to be responsive to the drug amphotericin B.^146^ Recently, Rouault et al used BLI to monitor leishmaniasis in real time in golden hamsters.^147^ They compared RLU signals from different organs with RT-PCR to quantify transcripts from both Leishmania and host cytokines. They found correlations between the transcriptional cytokine signatures and fluctuations in the amastigote burden in different tissues.

### Trypanosomal infection models

*Trypanosoma cruzi* is the causing agent of Chagas disease, a debilitating illness for humans. Studies have shown that the host cells of the reticuloendothelial and nervous systems, the muscles and adipocytes are the preferential targets not only in experimental animal models, but also in *T. cruzi*-infected patients. The use of BLI as a rapid and simple method for drug screening against Trypanosoma is increasing rapidly^148,149^.^150^ The pRIBOTEX expression vector (a derivative of pTEX) was introduced by Martı´nez-Calvillo as an efficient expression vector for construction and rapid selection of stably transfected *T. cruzi*.^151^ It was shown *T. cruzi* that had been transfected by pTEX expressing tandem tomato fluorescent protein genes (pTEX-Neo-tdTomato) could express bright red fluorescence at all stages of the life cycle.^152^ Canavaci et al. showed that BLI was useful for in-vitro and *in-vivo* high-throughput assays for the testing of new drugs against *T. cruzi*.^152^ BLI has been used in studies looking at drug screening, the mechanisms of cell invasion, genetic exchange among parasites, the roles of different factors in the outcome of infection and the differential tissue distribution of parasites in Trypanosome infected animal models. Myburgh et al.^150^ used BLI as a rapid drug screening method for following parasite clearance in the CNS stage of trypanosomiasis. The BLI results showed that the drugs melarsoprol and DB829 permanently eliminated all bioluminescent T. brucei from the mouse CNS. In another study on *T. brucei*, BLI was used to look at dissemination of the parasite in the animal model. The results demonstrated that *T. brucei* has a preferential tropism for the testes in male animals, and that clearance from testes was not as easy as clearance from abdominal cavity after drug treatment.^153^ For the first time, BLI was used as a non-invasive method to follow the infection of *Rhodinus prolixus* (the Trypansomal insect vector) by integrating the luciferase gene into the genome of the Dm28c clone of *T. cruzi*. The sensitivity and accuracy of BLI of the Dm28c-luc-infected digestive tract of the insects was demonstrated.^154^ Silva-Dos-Santos et al. used the *T. cruzi* Dm28c strain to study orally infected mice.^155^ They found that the nasomaxillary region was the initial site of parasite invasion in the host, while at later time points (7 and 21 days post-infection) the luminescent signal was more pronounced in the thorax, abdomen and genital regions, showing the parasites had disseminated to different organs

*T. vivax* is one of the most important parasites responsible for African trypanosomosis (Nagana or sleeping sickness), and is usually transmitted in a cyclical manner by *Glossina spp* (tsetse flies). D'Archivo et al constructed a West African IL1392 *T. vivax* strain stably expressing FLuc that was virulent in immunocompetent mice.^149^ They compared infection by the intraperitoneal and sub-cutaneous routes. When administered by the subcutaneous route, the parasite was retained for a few days in the skin fairly close to the inoculation site, where it multiplied before eventually passing into the bloodstream. When administered by IP injection systemic spread was much more rapid. *Ex vivo* bioluminescence analysis of isolated organs showed that the parasites had infiltrated into the spleen, liver and lungs, while brain infection was found in the very late stages.

### Toxoplasmosis infection models

In order to use BLI techniques for serial and non-lethal quantification of *Toxoplasma gondii *(*T. gondii*) *in-vivo*, type I and type II parasites expressing FLuc were developed.^156,157,158^ Light emission after intraperitoneal injection of D-Luciferin in mice, enabled investigation of the kinetics of infection with Toxoplasma in real-time. It was shown that there was a direct relationship between photon flux levels and the parasite load that allowed *in-vivo* quantification of the parasite burden.^157^

Saeij et al. used BLI for real-time monitoring of *in-vivo* growth, dissemination, and reactivation of strains of the protozoan parasite *T. gondii*. For this purpose, two *T. gondii* strains S23 (highly virulent) and S22 (low virulence) were engineered to stably express luciferase. While both groups of mice that were infected with S23 and S22 had the same initial growth in luminescence signals within a few days following infection, proliferation of strain S23 continued and led to severe disease, while in case of strain S22 the BLI signals become undetectable after a few days.^159^ It was claimed that the BLI method had advantages over other traditional methods such as plaque assays and quantitative PCR. Among these advantages, the first is that it includes the possibility of monitoring the kinetics and extension of disease progression in the same animal over time; the second is that a lower number of animals are needed; and thirdly that it is easier to perform. In this study, remarkable differences were observed in terms of organ dissemination between the mentioned strains, and high BLI signals in mice made it possible to monitor the progression of the infection non-invasively. The study also demonstrated the efficiency of BLI for monitoring anti-toxoplasma therapy and reactivation.^159^

Hitziger et al.^160^ used live-BLI to analyze the virulence of bioluminescent *T. gondii*. The results in a mouse model showed that the virulent RH *T. gondii* strain and the non-virulent ME49/PTG strain had the same initial dissemination, but in the case of virulent strain, a higher proliferation of parasites was observed. The study also demonstrated that there was a good correlation between light intensity and parasite numbers in spleen and testes. Furthermore, they did not observe any effect on susceptibility of mice to infection with these strains after disruption of various Toll-like receptors (TLR1, 2, 4, 6, or 9). A recent study^161^ investigated the effect of sequential exposure to single wall carbon nanotubes (SWCNT) via pharyngeal aspiration on the immune response of the infected mice against the *T. gondii*. BLI was used in this study to monitor the dissemination of *T. gondii*, and no differences were observed in terms of parasite distribution between infected mice and those pre-exposed to SWCNT before infection by *T. gondii*.

The first study to investigate the organ localization of acute *Toxoplasma encephalitis* infection in a mouse model was performed by Dellacasa-Lindberg et al.^156^ They used BLI to monitor the spatio-temporal localization of acute and reactivated *T. encephalitis* in mice. For this purpose, mice were inoculated i.p. with freshly prepared tachyzoites of the luciferase-expressing Toxoplasma strain and then followed daily by BLI. Ten days after inoculation when the bioluminescence signals had faded, asymptomatic mice were subjected to immunosuppression in order to reactivate Toxoplasma. Recrudescence mostly occurred in the CNS, and BLI enabled early detection and assessment of parasite reactivation.

## Viral infection models

BLI technology can be used to detect and monitor sites of viral infection and quantify viral replication in living animals.^162^ Some examples are given in Table 6. For this purpose, the recombinant viruses have been designed to express the luciferase enzyme. However this strategy is not very easy for RNA viruses, since stable insertion of an imaging reporter gene into the RNA virus genome is not feasible. The first report using viruses encoding luciferase together with BLI was published in 1988 by Rodriguez et al.^163^ These researchers introduced the Fluc gene into the vaccinia virus (VCAV) genome (under a VACV promoter) without affecting viral replication or pathogenesis in an animal model. The limits of detection were about one infected cell in a background of a million non-infected cells.^164^ Luker et al^165^ showed that replication of VACV was significantly faster in mice lacking receptors for type I interferons (IFN1R−/−) compared with wild-type mice, although both these mice eventually developed focal infections in the lungs and brain post intranasal inoculation. IFN1R−/− mice had more virus in the liver and spleen than wild-type mice, although death occurred at the same time point post-infection. They reported that the protective effects of type I interferons were mediated mainly via parenchymal cells rather than by hematopoietic cells as demonstrated by bone-marrow transplant studies.

**Table 6.** Viral infections monitored by BLI.

Disease	Virus	Host/Anatomical location	Type of luciferase	Model/ Treatment	Refs
Smallpox (orthopox virus)	Vaccinia WR strain	Female Balb/c mice inoculated IP Nasal cavity, lungs, spleen, liver	FLuc under immediate-early promoter (WRvFire)	Dryvax immunized and human intravenous vaccinia immunoglobulin (VIGIV) pre-treated mice were protected	^154^
Smallpox (orthopox virus)	Cowpox and monkeypox	Balb/c and CAST/Ei mice inoculated intranasally Head, chest and abdomen	FLuc	CAST/Ei mice were 100X more susceptible than Balb/c	^175^
Herpes simplex	HSV1 (KOS/dlux/oriL)	129Sv and IFNα/β/γ receptor KO mice Inoculated in footpad or cornea Spread to brain, lungs, liver, spleen, and body cavity	FLuc	IFN α/β receptor KO had worse infection than IFNγ KO	^168^
Viral encephalomyelitis	Sindbis alphavirus TRNSV virulent, NSV7 attenuated	Balb/c or C57BL/6 albino mice inoculated intracerebrally or SC; brain spinal cord	FLuc	C57BL/6 albino more susceptible than Balb/c	^170^
Flavivirus encephalitis	Japanese encephalitis virus	129Sv and IFN-R KO mice inoculated in intracranially or intraperitoneally; brain, intestine, spleen, liver, kidney and other abdominal organs	RLuc	Lack of type 1 IFN produces viscerotropism	^186^
Hepatitis B	HBV ayw serotype	Balb/c mice inoculated IV; Liver	FLuc with 4 promoters (C, S1, S2 and X) and 2 enhancers	Order of promoters C, X > S1, S2. Enhancers had no effect	^182^
Hepatitis C	HCV genotype 1b	Female C57BL/6 mice inoculated IV; liver	ANluc(NS5A/B)BCluc split FLuc fragments fused to interacting peptides with an intervening linker cleaved by NS3/4A protease	Signal was sensitive to NS3/4A protease and reduced by NS3/4A-specific shRNA and IFN-α	^178^
Influenza	A/California/04/2009 H1N1 virus (CA/09)	Ferrets innoculated intranasally; upper respiratory tract and lungs	NanoLuc	Can monitor intra-host dissemination, inter-host transmission and viral load	^187^

In another early report Lipshutz et al, created a luciferase expressing adeno-associated virus which was used with BLI in a mouse model.^166^

In another study, the role of interferons (IFN) in systemic herpes simplex infection (HSV-1) infection in mice model was investigated by BLI. This group showed that type I IFN receptors had a more important role in spread of HSV-1, and the absence of these receptors permitted the spread of this virus to parenchymal organs, lymph nodes and to neurons. However knockout of type II IFN receptors did not have the same effect and did not allow the systemic spread of HSV-1. Moreover the combined deletion of both type I and type II IFN receptors had a greater effect on encouraging the spread of virus to visceral organs, the nervous system and invariably led to death. In the last case, bioluminescence signals could be detected in the brain by 3 days post-infection.^167^

BLI has been used to monitor HSV-1 infection in living mice via luciferase expressing viruses, and the results showed that HSV-1 was disseminated throughout the mouse peritoneal cavity, footpads, eyes and brain. The infected mice were treated with valacyclovir, a potent HSV-1 inhibitor, and dose-dependent inhibition of the HSV-1 was demonstrated by both BLI data and viral titers.^168^ BLI was also used by Murphy et al^169^ to test the effect of interferon regulatory factors 3 and 7 (IRF-3 and IRF-7) on HSV-1 infection in IRF-3−/−, IRF-7−/− and double-knockout IRF3/7−/− (DKO) mice.

BLI was used in a murine model for monitoring the extent and dissemination of Sindbis virus (SV) replication over time without need to scarify infected mice. The BLI signals showed that the infection could spread from the olfactory epithelium to the CNS via retrograde axonal transport, or by direct penetration to the spinal cord.^170,171^ Sun et al^172^ constructed new expression vectors for two Old World alphaviruses (Sindbis and Chikungunya viruses) and two New World alphaviruses (Eastern and Venezuelan equine encephalitis viruses). These vectors contained either a large luciferase (FLuc; 1,650 nucleotides), or a small luciferase (NLuc; 513 nucleotides). The NLuc was more stable than FLuc during repeated rounds of infection and performed better for BLI in CD-1 mice infected with 1,000 PFU of SV injected subcutaneously in the ventral thorax region.

*Variola major* is an orthopoxvirus, which causes smallpox, and has attracted a high interest since it was declared to be a bioterrorist threat.^173,174^ The search for new vaccines against this agent needs accurate experimental models to predict lethality. In this sense, the estimation of viral burden based on BLI of several internal organs including the lungs resulted as the most accurate model to predict lethality, compared with the predictive power of animal weight reduction. Earl et al.^175^ studied monkeypox virus (an orthopoxvirus producing a smallpox-like zoonotic disease in humans). They compared the dissemination of monkeypox virus by BLI in inbred CAST/EiJ mice, and in the natural host (African dormice). In CAST/EiJ mice, a strong BLI signal was observed at the intranasal site of inoculation, and the virus disseminated rapidly to the lungs and abdominal organs, although these organs had less viral load. Compared to CAST/EiJ mice, African dormice showed a greater variability in the spread of the virus, a slower time course, less replication in the head and chest, with more replication in abdominal organs.

BLI mostly relies on construction of recombinant reporter viruses that can express firefly luciferase, in order to monitor viral replication and dissemination in live animals. The disadvantages of this approach such as limitations in analyzing multiple strains of the virus, need for further engineering of existing viral mutants, and possible attenuation of engineered reporter viruses in comparison to the parental viruses, has limited its applications. Luker et al developed a transgenic reporter mouse, which expressed firefly luciferase under control of the HSV-1 thymidine kinase (TK) promoter to facilitate BLI of HSV-1 infection. Infection with three different strains of HSV-1 (McKrae,17, and KOS) could be detected by BLI.^176^ Compared to other HSV-1 promoters such as ICP6 and ICP8, despite the lower basal activity, a higher induction of luminescence could be achieved in response to viral infection.

For determination of viral distribution and viral titers in traditional murine models, the animals need to be sacrificed, so new methods are needed in order to overcome this limitation.^177^ BLI and real-time PCR were used for monitoring the replication and tropism of HSV-1 virus in hematogenously infected mice. Both methods detected high viral loads in the ovaries and adrenal glands, however viral titers in nervous system were low. A good correlation was observed between the real-time PCR and BLI results. The results showed that BLI could be used to monitor HSV-1 hematogenous infection in living mice, by eliminating the need for sacrifice.^177^

Wang et al.,^178^ used BLI to monitor the activity of hepatitis C virus (HCV) that had been engineered to respond to the NS3/4A serine protease by a “split firefly luciferase complementation strategy”. The interacting peptides A and B were fused with the separated N-terminus and C-terminus amino acids of firefly luciferase, respectively, with cleavage sites for NS3/4A serine protease. It was shown that co-injection of a reporter plasmid containing a HCV NS3/4A serine protease with the engineered luciferase plasmid into mice, increased bioluminescence signals in comparison to control plasmids. Moreover, the results demonstrated the ability of this approach to screen NS3/4A inhibitors in mouse models.^178,179^ For real-time monitoring of two short hairpin (shRNAs) targeting the HCV core protein in living mice, the plasmid pGL3-attB-CoreFluc was constructed which encoded firefly luciferase fused downstream of the HCV core protein. BLI gave satisfactory results for real-time monitoring of HCV shRNA in living mice.^180^ Recently, *in-vivo* BLI of firefly luciferase-expressing NS3adenovirus was applied to investigate the clearance of HCV from the liver of transgenic humanized-HLA mice.^181^

BLI and hydrodynamic gene transfer technology were used to assess the activity of different hepatitis B virus (HBV) promoters (C, S1, S2, X) and enhancers.^182^ Results of this study indicated that, HBV enhancers had more prominent effects on three of the promoters (X, S1 and S2) *in-vivo* (mouse liver) than *in-vitro* (Hepa 1-6 cells) however these enhancers had no cooperative role in stimulating the HBV promoters. In another study, the persistence of transgene expression using HBV enhancers I and II combined with HBV core and X promoters was assessed by BLI. The HBV core and X promoter activity in hepatic cell lines could be stimulated by both HBV enhancers, and a constant high-level of gene expression was observed in mice, when either the HBV core promoter or the X promoter was linked to enhancer I and II.^183^ Recently, a new assay system for detection of HBV clearance in the liver was developed using BLI of a reporter gene (Fluc) after transferring linear HBV DNA and the Fluc gene into hepatocytes.^184^ The results showed a good correlation between viral clearance and control of luciferase expression in the infected hepatocytes. In one investigation, a non-invasive bioluminescence assay was applied in order to investigate the route of infectious hematopietic necrosis virus (IHNV) entry during natural infection of live fish. The results showed that the fin bases were the portal of entry of IHNV into fish.^185^

Li et al. used BLI to study neurotropic flaviviruses which can cause severe damages in the central and peripheral nerve systems. They constructed a recombinant Japanese encephalitis virus (JEV) expressing. They constructed a recombinant JEV virus expressing RLuc-JEV and inoculated mice either intraperitoneally or intracranially.^186^ In mice inoculated intraperitoneally, BLI signals could be detected not only from the brain but also from the abdominal organs. In addition, in mice inoculated intracranially, viral RNA measured by qRT-PCR directly correlated with the bioluminescence signal intensity. Mice deficient in IFN-1 receptors showed robust and prolonged viral replication in the abdominal organs.

Karlsson et al. studied influenza infection and transmission in ferrets using an engineered H1N1 influenza virus strain A/California/04/2009 encoding NanoLuc (NLuc).^187^ The group was able to detect bioluminescence signals from the respiratory tract and in less well-characterized extra-pulmonary sites. They could monitor intra-host dissemination, inter-host transmission, and quantify viral load which are highly relevant parameters for assessing the pandemic potential of this virus.

All these approaches using luciferase reporter viruses and longitudinal real-time monitoring, and quantitative analyses of viral infection using BLI have been immensely helpful for both pathogenesis studies, and for high-throughput screening for new anti-viral drugs which could be translated into clinical trials.^188^

## Food safety and plant infections

There are an increasing number of studies in the fields of biotechnology, environmental science and food safety that use BLI to detect and trace contamination by various microorganisms.

Karsi et al. developed Salmonella strains which contain pAKlux1 plasmid and constitutively express the luxCDABE operon.^189^ They studied the adherence of different strains to chicken skin and the effect of different washing protocols in removing the contamination.

Kassem et al studied Campylobacter contamination of chicken litter.^190^ They used shuttle plasmids that encoded luxCDABE into *C. jejuni* and *C. coli* to construct bioluminescent strains, that were then added to samples of litter-washings and dry litter collected from different cages for broiler chickens. They found that *C. jejuni* and *C. coli* survived for at least 20 days in reused (old) chicken litter while growth did not occur in clean (new) litter.

Rajeshekara and coworkers have used BLI to study the pathogenesis of “tomato canker”.^191,192^ *Clavibacter michiganensis subsp. michiganensis* (Cmm) is a rod-shaped, Gram-positive, aerobic actinomycete that causes bacterial canker in tomato plants. The canker causes impaired water transport and results in plant wilting, stunting, and death. The group used the modified transposon Tn1409 to chromosomally integrate the *P. luminescens* lux operon into Cmm^191^ and were able to study many aspects of the bacterial invasion process in tomato plants using BLI.

Maoz et al.^32^ used bioluminescent strains of *Yersinia enterocolitica* generated by transposon mutagenesis using a promoter-less, complete lux operon (luxCDABE) to allow direct BLI monitoring of *Y. enterocolitica* growth on cheeses stored at 10° C. The detection limit on cheese was 200 CFU/cm^2^. The bioluminescence signal from the B94 reporter strain was affected by the environment (NaCl concentration, temperature, and cheese type), as well as by its growth phase.

## Conclusions and future directions

BLI typically produces a single two-dimensional image of the entire animal, which can make it difficult to precisely localize sites of bioluminescence. Moreover BLI typically has only 1–3 mm spatial resolution, making it somewhat difficult to distinguish discrete sources of light arising from adjacent anatomical sites. There are ongoing efforts to develop 3D-hyperspectral BLI systems that will provide a tomographic approach and allow improvement in the spatial resolution of this modality.^193^

In the future it may be possible to generate cross-sectional BLI images with resolution, similar to X-ray, CT or MRI. Multi-modality small animal imaging systems that incorporate BLI with modalities that can be selected from a range including fluorescence, CT, MRI, PET, high resolution ultrasound and photoacoustic imaging are becoming increasingly available. While these systems have been developed mainly to carry out research in cancer therapy, their application to infectious disease models will undoubtedly soon follow.

For instance Collins et al.^194^ monitored the time course of a bioluminescent bacterial infection using composite 3D diffuse light imaging tomography with integrated μCT (DLIT-μCT) and generated a four dimensional (4D) movie of the infection cycle. They used bioluminescent *Citrobacter rodentium*, which causes self-limiting colitis in mice and non-invasive daily sessions of DLIT-μCT imaging was combined with bacterial CFU enumeration from feces over an 8 day period.

Since a lot of work in the area of microbial pathogenesis is concerned with investigating the host response to infectious disease, the ability to independently image the pathogens with BLI, and the host immune cells with fluorescence, PET or indeed with another color of bioluminescence would be extremely useful. For instance, lux-expressing bacteria emitting light at 480 nm can be combined with firefly or Renilla luciferase in the host cells emitting light at around 600 nm after application of the relevant substrate.

Despite the many advantages of BLI for monitoring of infectious disease, there are also some disadvantages and limitations. The genes encoding the luciferase enzymes may not be completely stable, and the signal may be lost with time especially when it is encoded by a plasmid. The requirement for sufficient O_2_ in the tissue to allow the light to be efficiently produced, may also be a limitation. Not only was this shown in the intestines, which are typically hypoxic, but other organs may also become hypoxic especially when a bacterial infection develops. The last limitation may occur when testing antimicrobial therapies. It is possible that the luciferase enzyme system is damaged by the therapy, but the bacterial ability to form colonies has not been abolished. Conversely the opposite is possible, where the bacterial ability to form colonies has been abolished, but residual luciferase activity is still able to produce some BLI signal.

It can be confidently predicted that the fast-growing field of BLI monitoring of infections will continue, and even accelerate as the imaging technology and the availability of bioluminescent organisms increases. Many commentators have remarked on the lack of development by the pharmaceutical industry of new antibiotics and innovative anti-microbial drugs.^195,196,197^ With the growth of antibiotic resistance predicted to become the single-biggest threat to global health^198^ this lack of research efforts on a big industrial scale will have to change, or the future of humanity will be in peril. Undoubtedly, the ability to screen libraries of compounds *in vivo* by non-invasive technologies like BLI will play an important role in this resurgence of antimicrobial research.

## References

[1] Zimmer M GFP: from jellyfish to the Nobel prize and beyond. Chem Soc Rev. 2009; 38:2823-32. https://doi.org/10.1039/b904023d. PMID:19771329 19771329

[2] Kricka LJ, Leach FR In memoriam Dr Marlene DeLuca. 1987 O. M. Smith Lecture. Firefly luciferase: mechanism of action, cloning and expression of the active enzyme. J Biolumin Chemilumin. 1989; 3:1-5. https://doi.org/10.1002/bio.1170030102. PMID:2652989 2652989

[3] Baldwin TO Firefly luciferase: the structure is known, but the mystery remains. Structure. 1996; 4:223-28. https://doi.org/10.1016/S0969-2126(96)00026-3. PMID:8805542 8805542

[4] Nakatani N, Hasegawa JY, Nakatsuji H Red light in chemiluminescence and yellow-green light in bioluminescence: color-tuning mechanism of firefly, Photinus pyralis, studied by the symmetry-adapted cluster-configuration interaction method. J Am Chem Soc. 2007; 129:8756-65. https://doi.org/10.1021/ja0611691. PMID:17585760 17585760

[5] Branchini BR, Southworth TL, Murtiashaw MH, Magyar RA, Gonzalez SA, Ruggiero MC, Stroh JG An alternative mechanism of bioluminescence color determination in firefly luciferase. Biochemistry. 2004; 43:7255-62. https://doi.org/10.1021/bi036175d. PMID:15182171 15182171

[6] Greer LF 3rd, Szalay AA Imaging of light emission from the expression of luciferases in living cells and organisms: a review. Luminescence. 2002; 17:43-74. https://doi.org/10.1002/bio.676. PMID:11816060 11816060

[7] Herring PJ Systematic distribution of bioluminescence in living organisms. J Biolumin Chemilumin. 1987; 1:147-63. https://doi.org/10.1002/bio.1170010303. PMID:3503524 3503524

[8] Chen AK, Latz MI, Frangos JA The use of dinoflagellate bioluminescence to characterize cell stimulation in bioreactors. Biotechnol Bioeng. 2003; 83:93-103. https://doi.org/10.1002/bit.10647. PMID:12740936 12740936

[9] Bechara EJ Bioluminescence: a fungal nightlight with an internal timer. Curr Biol. 2015; 25:R283-85. https://doi.org/10.1016/j.cub.2015.01.004. PMID:25829013 25829013

[10] Zhang Y, Bressler JP, Neal J, Lal B, Bhang HE, Laterra J, Pomper MG ABCG2/BCRP expression modulates D-Luciferin based bioluminescence imaging. Cancer Res. 2007; 67:9389-97. https://doi.org/10.1158/0008-5472.CAN-07-0944. PMID:17909048 17909048

[11] Dubikovskaya EA, Thorne SH, Pillow TH, Contag CH, Wender PA Overcoming multidrug resistance of small-molecule therapeutics through conjugation with releasable octaarginine transporters. Proc Natl Acad Sci U S A. 2008; 105:12128-33. https://doi.org/10.1073/pnas.0805374105. PMID:18713866 18713866 PMC2527877

[12] Heise K, Oppermann H, Meixensberger J, Gebhardt R, Gaunitz F Dual luciferase assay for secreted luciferases based on Gaussia and NanoLuc. Assay Drug Dev Technol. 2013; 11:244-52. https://doi.org/10.1089/adt.2013.509. PMID:23679848 23679848

[13] Francis KP, Yu J, Bellinger-Kawahara C, Joh D, Hawkinson MJ, Xiao G, Purchio TF, Caparon MG, Lipsitch M, Contag PR Visualizing pneumococcal infections in the lungs of live mice using bioluminescent Streptococcus pneumoniae transformed with a novel gram-positive lux transposon. Infect Immun. 2001; 69:3350-58. https://doi.org/10.1128/IAI.69.5.3350-3358.2001. PMID:11292758 11292758 PMC98294

[14] England CG, Ehlerding EB, Cai W NanoLuc: A Small Luciferase Is Brightening Up the Field of Bioluminescence. Bioconjug Chem. 2016; 27:1175-87. https://doi.org/10.1021/acs.bioconjchem.6b00112. PMID:27045664 27045664 PMC4871753

[15] Hutchens M, Luker GD Applications of bioluminescence imaging to the study of infectious diseases. Cell Microbiol. 2007; 9:2315-22. https://doi.org/10.1111/j.1462-5822.2007.00995.x. PMID:17587328 17587328

[16] Gahan CG The bacterial lux reporter system: applications in bacterial localisation studies. Curr Gene Ther. 2012; 12:12-19. https://doi.org/10.2174/156652312799789244. PMID:22263920 22263920

[17] Contag CH, Contag PR, Mullins JI, Spilman SD, Stevenson DK, Benaron DA Photonic detection of bacterial pathogens in living hosts. Mol Microbiol. 1995; 18:593-603. https://doi.org/10.1111/j.1365-2958.1995.mmi_18040593.x. PMID:8817482 8817482

[18] Vecchio D, Dai T, Huang L, Fantetti L, Roncucci G, Hamblin MR Antimicrobial photodynamic therapy with RLP068 kills methicillin-resistant Staphylococcus aureus and improves wound healing in a mouse model of infected skin abrasion PDT with RLP068/Cl in infected mouse skin abrasion. J Biophotonics. 2013; 6:733-42. https://doi.org/10.1002/jbio.201200121. PMID:22987338 22987338 PMC3594622

[19] Ryan PL, Christiansen DL, Hopper RM, Walters FK, Moulton K, Curbelo J, Greene JM, Willard ST Horse species symposium: a novel approach to monitoring pathogen progression during uterine and placental infection in the mare using bioluminescence imaging technology and lux-modified bacteria. J Anim Sci. 2011; 89:1541-51. https://doi.org/10.2527/jas.2010-3629. PMID:21239661 21239661

[20] Wiles S, Clare S, Harker J, Huett A, Young D, Dougan G, Frankel G Organ specificity, colonization and clearance dynamics *in vivo* following oral challenges with the murine pathogen Citrobacter rodentium. Cell Microbiol. 2004; 6:963-72. https://doi.org/10.1111/j.1462-5822.2004.00414.x. PMID:15339271 15339271

[21] Short KR, Diavatopoulos DA, Reading PC, Brown LE, Rogers KL, Strugnell RA, Wijburg OL Using bioluminescent imaging to investigate synergism between Streptococcus pneumoniae and influenza A virus in infant mice. J Vis Exp. 2011; 50: 2357 10.3791/2357. PMID: 21525841 PMC3169275 21525841

[22] Zhang T, Li SY, Converse PJ, Almeida DV, Grosset JH, Nuermberger EL Using bioluminescence to monitor treatment response in real time in mice with Mycobacterium ulcerans infection. Antimicrob Agents Chemother. 2011; 55:56-61. https://doi.org/10.1128/AAC.01260-10. PMID:21078940 21078940 PMC3019670

[23] Jacobsen ID, Lüttich A, Kurzai O, Hube B, Brock M In vivo imaging of disseminated murine Candida albicans infection reveals unexpected host sites of fungal persistence during antifungal therapy. J Antimicrob Chemother. 2014; 69:2785-96. https://doi.org/10.1093/jac/dku198. PMID:24951534 24951534

[24] Mosci P, Gabrielli E, Luciano E, Perito S, Cassone A, Pericolini E, Vecchiarelli A Involvement of IL-17A in preventing the development of deep-seated candidiasis from oropharyngeal infection. Microbes Infect. 2014; 16:678-89. https://doi.org/10.1016/j.micinf.2014.06.007. PMID:24980544 24980544

[25] Mosci P, Pericolini E, Gabrielli E, Kenno S, Perito S, Bistoni F, d'Enfert C, Vecchiarelli A A novel bioluminescence mouse model for monitoring oropharyngeal candidiasis in mice. Virulence. 2013; 4:250-54. https://doi.org/10.4161/viru.23529. PMID:23334179 23334179 PMC3711983

[26] Pietrella D, Rachini A, Pines M, Pandey N, Mosci P, Bistoni F, d'Enfert C, Vecchiarelli A Th17 cells and IL-17 in protective immunity to vaginal candidiasis. PLoS One. 2011; 6:e22770.https://doi.org/10.1371/journal.pone.0022770. PMID:21818387 21818387 PMC3144947

[27] Ibrahim-Granet O, Jouvion G, Hohl TM, Droin-Bergère S, Philippart F, Kim OY, Adib-Conquy M, Schwendener R, Cavaillon JM, Brock M In vivo bioluminescence imaging and histopathopathologic analysis reveal distinct roles for resident and recruited immune effector cells in defense against invasive aspergillosis. BMC Microbiol. 2010; 10:105.https://doi.org/10.1186/1471-2180-10-105. PMID:20377900 20377900 PMC2859869

[28] Hamblin MR, O'Donnell DA, Murthy N, Contag CH, Hasan T Rapid control of wound infections by targeted photodynamic therapy monitored by *in vivo* bioluminescence imaging. Photochem Photobiol. 2002; 75:51-57. https://doi.org/10.1562/0031-8655(2002)075%3c0051:RCOWIB%3e2.0.CO;2. PMID:11837327 11837327

[29] Dai T, Tegos GP, Lu Z, Huang L, Zhiyentayev T, Franklin MJ, Baer DG, Hamblin MR Photodynamic therapy for Acinetobacter baumannii burn infections in mice. Antimicrob Agents Chemother. 2009; 53:3929-34. https://doi.org/10.1128/AAC.00027-09. PMID:19564369 19564369 PMC2737832

[30] Gad F, Zahra T, Francis KP, Hasan T, Hamblin MR Targeted photodynamic therapy of established soft-tissue infections in mice. Photochem Photobiol Sci. 2004; 3:451-58. https://doi.org/10.1039/b311901g. PMID:15122362 15122362 PMC3071693

[31] Garcez AS, Ribeiro MS, Tegos GP, Núñez SC, Jorge AO, Hamblin MR Antimicrobial photodynamic therapy combined with conventional endodontic treatment to eliminate root canal biofilm infection. Lasers Surg Med. 2007; 39:59-66. https://doi.org/10.1002/lsm.20415. PMID:17066481 17066481 PMC3071045

[32] Maoz A, Mayr R, Bresolin G, Neuhaus K, Francis KP, Scherer S Sensitive in situ monitoring of a recombinant bioluminescent Yersinia enterocolitica reporter mutant in real time on Camembert cheese. Appl Environ Microbiol. 2002; 68:5737-40. https://doi.org/10.1128/AEM.68.11.5737-5740.2002. PMID:12406772 12406772 PMC129901

[33] Enjalbert B, Rachini A, Vediyappan G, Pietrella D, Spaccapelo R, Vecchiarelli A, Brown AJ, d'Enfert C A multifunctional, synthetic Gaussia princeps luciferase reporter for live imaging of Candida albicans infections. Infect Immun. 2009; 77:4847-58. https://doi.org/10.1128/IAI.00223-09. PMID:19687206 19687206 PMC2772526

[34] Jett BD, Hatter KL, Huycke MM, Gilmore MS Simplified agar plate method for quantifying viable bacteria. Biotechniques. 1997; 23:648-50. PMID:9343684 9343684 10.2144/97234bm22

[35] Dai T, Kharkwal GB, Tanaka M, Huang YY, Bil de Arce VJ, Hamblin MR Animal models of external traumatic wound infections. Virulence. 2011; 2:296-315. https://doi.org/10.4161/viru.2.4.16840. PMID:21701256 21701256 PMC3173676

[36] Demidova TN, Gad F, Zahra T, Francis KP, Hamblin MR Monitoring photodynamic therapy of localized infections by bioluminescence imaging of genetically engineered bacteria. J Photochem Photobiol B. 2005; 81:15-25. https://doi.org/10.1016/j.jphotobiol.2005.05.007. PMID:16040251 16040251 PMC3071690

[37] Dai T, Huang YY, Hamblin MR Photodynamic therapy for localized infections–state of the art. Photodiagnosis Photodyn Ther. 2009; 6:170-188. https://doi.org/10.1016/j.pdpdt.2009.10.008. PMID:19932449 19932449 PMC2811240

[38] Hamblin MR, Hasan T Photodynamic therapy: a new antimicrobial approach to infectious disease? Photochem Photobiol Sci. 2004; 3:436-50. https://doi.org/10.1039/b311900a. PMID:15122361 15122361 PMC3071049

[39] Hamblin MR, Zahra T, Contag CH, McManus AT, Hasan T Optical monitoring and treatment of potentially lethal wound infections *in vivo*. J Infect Dis. 2003; 187:1717-25. https://doi.org/10.1086/375244. PMID:12751029 12751029 PMC3441051

[40] Lu Z, Dai T, Huang L, Kurup DB, Tegos GP, Jahnke A, Wharton T, Hamblin MR Photodynamic therapy with a cationic functionalized fullerene rescues mice from fatal wound infections. Nanomedicine (Lond). 2010; 5:1525-33. https://doi.org/10.2217/nnm.10.98. PMID:21143031 21143031 PMC3047412

[41] Dai T, Tegos GP, Zhiyentayev T, Mylonakis E, Hamblin MR Photodynamic therapy for methicillin-resistant Staphylococcus aureus infection in a mouse skin abrasion model. Lasers Surg Med. 2010; 42:38-44. https://doi.org/10.1002/lsm.20887. PMID:20077489 20077489 PMC2820267

[42] Dai T, Bil de Arce VJ, Tegos GP, Hamblin MR Blue dye and red light, a dynamic combination for prophylaxis and treatment of cutaneous Candida albicans infections in mice. Antimicrob Agents Chemother. 2011; 55:5710-17. https://doi.org/10.1128/AAC.05404-11. PMID:21930868 21930868 PMC3232820

[43] Heunis TD, Smith C, Dicks LM Evaluation of a nisin-eluting nanofiber scaffold to treat Staphylococcus aureus-induced skin infections in mice. Antimicrob Agents Chemother. 2013; 57:3928-35. https://doi.org/10.1128/AAC.00622-13. PMID:23733456 23733456 PMC3719752

[44] Hertlein T, Sturm V, Jakob P, Ohlsen K 19F magnetic resonance imaging of perfluorocarbons for the evaluation of response to antibiotic therapy in a Staphylococcus aureus infection model. PLoS One. 2013; 8:e64440.https://doi.org/10.1371/journal.pone.0064440. PMID:23724049 23724049 PMC3665837

[45] Engelsman AF, van Dam GM, van der Mei HC, Busscher HJ, Ploeg RJ In vivo evaluation of bacterial infection involving morphologically different surgical meshes. Ann Surg. 2010; 251:133-37. https://doi.org/10.1097/SLA.0b013e3181b61d9a. PMID:19864938 19864938

[46] Peetermans M, Vanassche T, Liesenborghs L, Claes J, Vande Velde G, Kwiecinksi J, Jin T, De Geest B, Hoylaerts MF, Lijnen RH, et al. Plasminogen activation by staphylokinase enhances local spreading of *S. aureus* in skin infections. BMC Microbiol. 2014; 14:310.https://doi.org/10.1186/s12866-014-0310-7. PMID:25515118 25515118 PMC4274676

[47] Huang L, Dai T, Xuan Y, Tegos GP, Hamblin MR Synergistic combination of chitosan acetate with nanoparticle silver as a topical antimicrobial: efficacy against bacterial burn infections. Antimicrob Agents Chemother. 2011; 55:3432-38. https://doi.org/10.1128/AAC.01803-10. PMID:21502618 21502618 PMC3122390

[48] Ragàs X, Dai T, Tegos GP, Agut M, Nonell S, Hamblin MR Photodynamic inactivation of Acinetobacter baumannii using phenothiazinium dyes: *in vitro* and *in vivo* studies. Lasers Surg Med. 2010; 42:384-90. https://doi.org/10.1002/lsm.20922. PMID:20583252 20583252 PMC2935797

[49] Ragàs X, Sánchez-García D, Ruiz-González R, Dai T, Agut M, Hamblin MR, Nonell S Cationic porphycenes as potential photosensitizers for antimicrobial photodynamic therapy. J Med Chem. 2010; 53:7796-803. https://doi.org/10.1021/jm1009555. PMID:20936792 20936792 PMC2981434

[50] Dirschl DR, Almekinders LC Osteomyelitis. Common causes and treatment recommendations. Drugs. 1993; 45:29-43. https://doi.org/10.2165/00003495-199345010-00004. PMID:7680983 7680983

[51] Shi S, Zhang X Interaction of Staphylococcus aureus with osteoblasts (Review). Exp Ther Med. 2012; 3:367-70. https://doi.org/10.3892/etm.2011.423. PMID:22969897 22969897 PMC3438663

[52] Li D, Gromov K, Søballe K, Puzas JE, O'Keefe RJ, Awad H, Drissi H, Schwarz EM Quantitative mouse model of implant-associated osteomyelitis and the kinetics of microbial growth, osteolysis, and humoral immunity. J Orthop Res. 2008; 26:96-105. https://doi.org/10.1002/jor.20452. PMID:17676625 17676625 PMC2701346

[53] Hardy J, Chu P, Contag CH Foci of Listeria monocytogenes persist in the bone marrow. Dis Model Mech. 2009; 2:39-46. https://doi.org/10.1242/dmm.000836. PMID:19132117 19132117 PMC2615163

[54] Funao H, Ishii K, Nagai S, Sasaki A, Hoshikawa T, Aizawa M, Okada Y, Chiba K, Koyasu S, Toyama Y, et al. Establishment of a real-time, quantitative, and reproducible mouse model of Staphylococcus osteomyelitis using bioluminescence imaging. Infect Immun. 2012; 80:733-41. https://doi.org/10.1128/IAI.06166-11. PMID:22104103 22104103 PMC3264289

[55] Bisland SK, Chien C, Wilson BC, Burch S Pre-clinical *in vitro* and *in vivo* studies to examine the potential use of photodynamic therapy in the treatment of osteomyelitis. Photochem Photobiol Sci. 2006; 5:31-38. https://doi.org/10.1039/B507082A. PMID:16395425 16395425

[56] Wainwright M, Byrne MN, Gattrell MA Phenothiazinium-based photobactericidal materials. J Photochem Photobiol B. 2006; 84:227-30. https://doi.org/10.1016/j.jphotobiol.2006.03.002. PMID:16713280 16713280

[57] Bellnier DA, Greco WR, Loewen GM, Nava H, Oseroff AR, Dougherty TJ Clinical pharmacokinetics of the PDT photosensitizers porfimer sodium (Photofrin), 2-[1-hexyloxyethyl]-2-devinyl pyropheophorbide-a (Photochlor) and 5-ALA-induced protoporphyrin IX. Lasers Surg Med. 2006; 38:439-44. https://doi.org/10.1002/lsm.20340. PMID:16634075 16634075

[58] Wickham ME, Brown NF, Provias J, Finlay BB, Coombes BK Oral infection of mice with Salmonella enterica serovar Typhimurium causes meningitis and infection of the brain. BMC Infect Dis. 2007; 7:65.https://doi.org/10.1186/1471-2334-7-65. PMID:17597539 17597539 PMC1925087

[59] Monack DM, Bouley DM, Falkow S Salmonella typhimurium persists within macrophages in the mesenteric lymph nodes of chronically infected Nramp1+/+ mice and can be reactivated by IFNgamma neutralization. J Exp Med. 2004; 199:231-41. https://doi.org/10.1084/jem.20031319. PMID:14734525 14734525 PMC2211772

[60] Burns-Guydish SM, Olomu IN, Zhao H, Wong RJ, Stevenson DK, Contag CH Monitoring age-related susceptibility of young mice to oral Salmonella enterica serovar Typhimurium infection using an *in vivo* murine model. Pediatr Res. 2005; 58:153-58. https://doi.org/10.1203/01.PDR.0000157725.44213.C4. PMID:15774831 15774831

[61] Burns-Guydish SM, Zhao H, Stevenson DK, Contag CH The potential Salmonella aroA- vaccine strain is safe and effective in young BALB/c mice. Neonatology. 2007; 91:114-20. https://doi.org/10.1159/000097128. PMID:17344661 17344661

[62] Özkaya H, Akcan AB, Aydemir G, Aydinöz S, Razia Y, Gammon ST, McKinney J Salmonella typhimurium infections in BALB/c mice: a comparison of tissue bioluminescence, tissue cultures and mice clinical scores. New Microbiol. 2012; 35:53-59. PMID:22378553 22378553

[63] Rhee KJ, Cheng H, Harris A, Morin C, Kaper JB, Hecht G Determination of spatial and temporal colonization of enteropathogenic E. coli and enterohemorrhagic E. coli in mice using bioluminescent *in vivo* imaging. Gut Microbes. 2011; 2:34-41. https://doi.org/10.4161/gmic.2.1.14882. PMID:21637016 21637016 PMC3225795

[64] Sun J, Wang XD, Liu H, Xu JG Ketamine suppresses endotoxin-induced NF-kappaB activation and cytokines production in the intestine. Acta Anaesthesiol Scand. 2004; 48:317-21. https://doi.org/10.1111/j.0001-5172.2004.0312.x. PMID:14982564 14982564

[65] Welters ID, Hafer G, Menzebach A, Mühling J, Neuhäuser C, Browning P, Goumon Y Ketamine inhibits transcription factors activator protein 1 and nuclear factor-kappaB, interleukin-8 production, as well as CD11b and CD16 expression: studies in human leukocytes and leukocytic cell lines. Anesth Analg. 2010; 110:934-41. https://doi.org/10.1213/ANE.0b013e3181c95cfa. PMID:20185670 20185670

[66] La Rosa SL, Diep DB, Nes IF, Brede DA Construction and application of a luxABCDE reporter system for real-time monitoring of Enterococcus faecalis gene expression and growth. Appl Environ Microbiol. 2012; 78:7003-11. https://doi.org/10.1128/AEM.02018-12. PMID:22843522 22843522 PMC3457518

[67] La Rosa SL, Casey PG, Hill C, Diep DB, Nes IF, Brede DA In vivo assessment of growth and virulence gene expression during commensal and pathogenic lifestyles of luxABCDE-tagged Enterococcus faecalis strains in murine gastrointestinal and intravenous infection models. Appl Environ Microbiol. 2013; 79:3986-97. https://doi.org/10.1128/AEM.00831-13. PMID:23603680 23603680 PMC3697570

[68] Parajuli NP, Maharjan P, Parajuli H, Joshi G, Paudel D, Sayami S, Khanal PR High rates of multidrug resistance among uropathogenic Escherichia coli in children and analyses of ESBL producers from Nepal. Antimicrob Resist Infect Control. 2017; 6:9.https://doi.org/10.1186/s13756-016-0168-6. PMID:28096977 28096977 PMC5225645

[69] Tofte N, Nielsen AC, Trøstrup H, Andersen CB, Von Linstow M, Hansen B, Biering-Sørensen F, Høiby N, Moser C Chronic urinary tract infections in patients with spinal cord lesions – biofilm infection with need for long-term antibiotic treatment. APMIS. 2017; 125:385-91. https://doi.org/10.1111/apm.12685. PMID:28407430 28407430

[70] Balsara ZR, Ross SS, Dolber PC, Wiener JS, Tang Y, Seed PC Enhanced susceptibility to urinary tract infection in the spinal cord-injured host with neurogenic bladder. Infect Immun. 2013; 81:3018-26. https://doi.org/10.1128/IAI.00255-13. PMID:23753628 23753628 PMC3719561

[71] Mulvey MA, Schilling JD, Hultgren SJ Establishment of a persistent Escherichia coli reservoir during the acute phase of a bladder infection. Infect Immun. 2001; 69:4572-79. https://doi.org/10.1128/IAI.69.7.4572-4579.2001. PMID:11402001 11402001 PMC98534

[72] Andrew PW, Roberts IS Construction of a bioluminescent mycobacterium and its use for assay of antimycobacterial agents. J Clin Microbiol. 1993; 31:2251-54. PMID:8408541 8408541 10.1128/jcm.31.9.2251-2254.1993 PMC265743

[73] Deb DK, Srivastava KK, Srivastava R, Srivastava BS Bioluminescent Mycobacterium aurum expressing firefly luciferase for rapid and high throughput screening of antimycobacterial drugs *in vitro* and in infected macrophages. Biochem Biophys Res Commun. 2000; 279:457-61. https://doi.org/10.1006/bbrc.2000.3957. PMID:11118308 11118308

[74] Arain TM, Resconi AE, Singh DC, Stover CK Reporter gene technology to assess activity of antimycobacterial agents in macrophages. Antimicrob Agents Chemother. 1996; 40:1542-44. PMID:8726035 8726035 10.1128/aac.40.6.1542 PMC163365

[75] Andreu N, Fletcher T, Krishnan N, Wiles S, Robertson BD Rapid measurement of antituberculosis drug activity *in vitro* and in macrophages using bioluminescence. J Antimicrob Chemother. 2012; 67:404-14. https://doi.org/10.1093/jac/dkr472. PMID:22101217 22101217 PMC3254196

[76] Andreu N, Zelmer A, Sampson SL, Ikeh M, Bancroft GJ, Schaible UE, Wiles S, Robertson BD Rapid *in vivo* assessment of drug efficacy against Mycobacterium tuberculosis using an improved firefly luciferase. J Antimicrob Chemother. 2013; 68:2118-27. https://doi.org/10.1093/jac/dkt155. PMID:23633686 23633686 PMC3743513

[77] Andreu N, Zelmer A, Fletcher T, Elkington PT, Ward TH, Ripoll J, Parish T, Bancroft GJ, Schaible U, Robertson BD, et al. Optimisation of bioluminescent reporters for use with mycobacteria. PLoS One. 2010; 5:e10777.https://doi.org/10.1371/journal.pone.0010777. PMID:20520722 20520722 PMC2875389

[78] Zhang T, Li SY, Nuermberger EL Autoluminescent Mycobacterium tuberculosis for rapid, real-time, non-invasive assessment of drug and vaccine efficacy. PLoS One. 2012; 7:e29774.https://doi.org/10.1371/journal.pone.0029774. PMID:22253776 22253776 PMC3256174

[79] Siqueira JF Jr Endodontic infections: concepts, paradigms, and perspectives. Oral Surg Oral Med Oral Pathol Oral Radiol Endod. 2002; 94:281-93. https://doi.org/10.1067/moe.2002.126163. PMID:12324780 12324780

[80] Garcez AS, Nunez SC, Lage-Marques JL, Hamblin MR, Ribeiro MS Photonic real-time monitoring of bacterial reduction in root canals by genetically engineered bacteria after chemomechanical endodontic therapy. Braz Dent J. 2007; 18:202-07. https://doi.org/10.1590/S0103-64402007000300005. PMID:18176710 18176710 PMC2940265

[81] Sedgley CM, Nagel AC, Hall D, Applegate B Influence of irrigant needle depth in removing bioluminescent bacteria inoculated into instrumented root canals using real-time imaging *in vitro*. Int Endod J. 2005; 38:97-104. https://doi.org/10.1111/j.1365-2591.2004.00906.x. PMID:15667631 15667631

[82] Sedgley C, Applegate B, Nagel A, Hall D Real-time imaging and quantification of bioluminescent bacteria in root canals *in vitro*. J Endod. 2004; 30:893-98. https://doi.org/10.1097/01.DON.0000132299.02265.6C. PMID:15564873 15564873

[83] Sabino CP, Garcez AS, Núñez SC, Ribeiro MS, Hamblin MR Real-time evaluation of two light delivery systems for photodynamic disinfection of Candida albicans bio- film in curved root canals. Lasers Med Sci. 2014; 6: 1657–65. https://doi:10.1007/s10103-014-1629-x. PMID:25060900 25060900 PMC4305509

[84] Bray M, Lawler J, Paragas J, Jahrling PB, Mollura DJ Molecular imaging of influenza and other emerging respiratory viral infections. J Infect Dis. 2011; 203:1348-59. https://doi.org/10.1093/infdis/jir038. PMID:21422476 21422476 PMC3080905

[85] Smith MW, Schmidt JE, Rehg JE, Orihuela CJ, McCullers JA Induction of pro- and anti-inflammatory molecules in a mouse model of pneumococcal pneumonia after influenza. Comp Med. 2007; 57:82-89. PMID:17348295 17348295 PMC2736785

[86] Henken S, Bohling J, Ogunniyi AD, Paton JC, Salisbury VC, Welte T, Maus UA Evaluation of biophotonic imaging to estimate bacterial burden in mice infected with highly virulent compared to less virulent Streptococcus pneumoniae serotypes. Antimicrob Agents Chemother. 2010; 54:3155-60. https://doi.org/10.1128/AAC.00310-10. PMID:20530224 20530224 PMC2916300

[87] Debarbieux L, Leduc D, Maura D, Morello E, Criscuolo A, Grossi O, Balloy V, Touqui L Bacteriophages can treat and prevent Pseudomonas aeruginosa lung infections. J Infect Dis. 2010; 201:1096-104. https://doi.org/10.1086/651135. PMID:20196657 20196657

[88] Giebink GS, Payne EE, Mills EL, Juhn SK, Quie PG Experimental otitis media due to Streptococcus pneumoniae: immunopathogenic response in the chinchilla. J Infect Dis. 1976; 134:595-604. https://doi.org/10.1093/infdis/134.6.595. PMID:12236 12236

[89] Lusis PI, Soltys MA Immunization of mice and chinchillas against Pseudomonas aeruginosa. Can J Comp Med. 1971; 35:60-66. PMID:4251417 4251417 PMC1319542

[90] Doyle WJ, Supance JS, Marshak G, Cantekin EI, Bluestone CD, Rohn DD An animal model of acute otitis media consequent to beta-lactamase-producing nontypable Haemophilus influenzae. Otolaryngol Head Neck Surg. 1982; 90:831-36. https://doi.org/10.1177/019459988209000627. PMID:10994438 10994438

[91] Chung MH, Enrique R, Lim DJ, De Maria TF Moraxella (Branhamella) catarrhalis-induced experimental otitis media in the chinchilla. Acta Otolaryngol. 1994; 114:415-22. https://doi.org/10.3109/00016489409126080. PMID:7976314 7976314

[92] Post JC Direct evidence of bacterial biofilms in otitis media. Laryngoscope. 2001; 111:2083-94. https://doi.org/10.1097/00005537-200112000-00001. PMID:11802002 11802002

[93] Giebink GS Otitis media: the chinchilla model. Microb Drug Resist. 1999; 5:57-72. https://doi.org/10.1089/mdr.1999.5.57. PMID:10332723 10332723

[94] Chaney EJ, Nguyen CT, Boppart SA Novel method for non-invasive induction of a middle-ear biofilm in the rat. Vaccine. 2011; 29:1628-33. https://doi.org/10.1016/j.vaccine.2010.12.076. PMID:21211589 21211589 PMC3200228

[95] Novotny LA, Mason KM, Bakaletz LO Development of a chinchilla model to allow direct, continuous, biophotonic imaging of bioluminescent nontypeable Haemophilus influenzae during experimental otitis media. Infect Immun. 2005; 73:609-11. https://doi.org/10.1128/IAI.73.1.609-611.2005. PMID:15618201 15618201 PMC538955

[96] Peltola VT, Boyd KL, McAuley JL, Rehg JE, McCullers JA Bacterial sinusitis and otitis media following influenza virus infection in ferrets. Infect Immun. 2006. 74: 2562-67. https://doi.org/10.1128/IAI.74.5.2562-2567.2006. PMID: 16622191 16622191 PMC1459735

[97] Saez-Llorens X, McCracken GH Jr Bacterial meningitis in neonates and children. Infect Dis Clin North Am. 1990; 4:623-44. PMID:2277192 2277192

[98] Sjolinder H, Jonsson AB In vivo imaging of meningococcal disease dynamics. Methods Mol Biol. 2012; 799:153-68. https://doi.org/10.1007/978-1-61779-346-2_10. PMID:21993645 21993645

[99] Sjolinder H, Jonsson AB Imaging of disease dynamics during meningococcal sepsis. PLoS One. 2007; 2:e241.https://doi.org/10.1371/journal.pone.0000241. PMID:17311106 17311106 PMC1797199

[100] Mook-Kanamori BB, Rouse MS, Kang CI, van de Beek D, Steckelberg JM, Patel R Daptomycin in experimental murine pneumococcal meningitis. BMC Infect Dis. 2009; 9:50.https://doi.org/10.1186/1471-2334-9-50. PMID:19405978 19405978 PMC2685802

[101] Kadurugamuwa JL, Modi K, Coquoz O, Rice B, Smith S, Contag PR, Purchio T Reduction of astrogliosis by early treatment of pneumococcal meningitis measured by simultaneous imaging, *in vivo*, of the pathogen and host response. Infect Immun. 2005; 73:7836-43. https://doi.org/10.1128/IAI.73.12.7836-7843.2005. PMID:16299273 16299273 PMC1307043

[102] Yadav KK, Mandal AK, Sen IK, Chakraborti S, Islam SS, Chakraborty R Flocculating property of extracellular polymeric substances produced by a biofilm-forming bacterium Acinetobacter junii BB1A. Appl Biochem Biotechnol. 2012; 168:1621-34. https://doi.org/10.1007/s12010-012-9883-5. PMID:22968590 22968590

[103] Alavi MR, Stojadinovic A, Izadjoo MJ An overview of biofilm and its detection in clinical samples. J Wound Care. 2012; 21:376-83. https://doi.org/10.12968/jowc.2012.21.8.376. PMID:22885310 22885310

[104] Mombelli A, Decaillet F The characteristics of biofilms in peri-implant disease. J Clin Periodontol. 2011; 38(Suppl 11):203-13. https://doi.org/10.1111/j.1600-051X.2010.01666.x. PMID:21323716 21323716

[105] Lönn-Stensrud J, Landin MA, Benneche T, Petersen FC, Scheie AA Furanones, potential agents for preventing Staphylococcus epidermidis biofilm infections? J Antimicrob Chemother. 2009; 63:309-16. https://doi.org/10.1093/jac/dkn501. PMID:19098295 19098295

[106] Pribaz JR, Bernthal NM, Billi F, Cho JS, Ramos RI, Guo Y, Cheung AL, Francis KP, Miller LS Mouse model of chronic post-arthroplasty infection: noninvasive *in vivo* bioluminescence imaging to monitor bacterial burden for long-term study. J Orthop Res. 2012; 30:335-40. https://doi.org/10.1002/jor.21519. PMID:21837686 21837686 PMC3217109

[107] Engelsman AF, van der Mei HC, Francis KP, Busscher HJ, Ploeg RJ, van Dam GM Real time noninvasive monitoring of contaminating bacteria in a soft tissue implant infection model. J Biomed Mater Res B Appl Biomater. 2009; 88:123-29. https://doi.org/10.1002/jbm.b.31158. PMID:18618733 18618733

[108] Niska JA, Shahbazian JH, Ramos RI, Pribaz JR, Billi F, Francis KP, Miller LS Daptomycin and tigecycline have broader effective dose ranges than vancomycin as prophylaxis against a Staphylococcus aureus surgical implant infection in mice. Antimicrob Agents Chemother. 2012; 56:2590-97. https://doi.org/10.1128/AAC.06291-11. PMID:22371896 22371896 PMC3346658

[109] Chauhan A, Lebeaux D, Decante B, Kriegel I, Escande MC, Ghigo JM, Beloin C A rat model of central venous catheter to study establishment of long-term bacterial biofilm and related acute and chronic infections. PLoS One. 2012; 7:e37281.https://doi.org/10.1371/journal.pone.0037281. PMID:22615964 22615964 PMC3353920

[110] Xiong YQ, Willard J, Kadurugamuwa JL, Yu J, Francis KP, Bayer AS Real-time *in vivo* bioluminescent imaging for evaluating the efficacy of antibiotics in a rat Staphylococcus aureus endocarditis model. Antimicrob Agents Chemother. 2005; 49:380-387. https://doi.org/10.1128/AAC.49.1.380-387.2005. PMID:15616318 15616318 PMC538900

[111] Soll DR The regulation of cellular differentiation in the dimorphic yeast Candida albicans. Bioessays. 1986; 5:5-11. https://doi.org/10.1002/bies.950050103. PMID:3539113 3539113

[112] Soll DR, Morrow B, Srikantha T High-frequency phenotypic switching in Candida albicans. Trends Genet. 1993; 9:61-65. https://doi.org/10.1016/0168-9525(93)90189-O. PMID:8456504 8456504

[113] Srikantha T, Klapach A, Lorenz WW, Tsai LK, Laughlin LA, Gorman JA, Soll DR The sea pansy Renilla reniformis luciferase serves as a sensitive bioluminescent reporter for differential gene expression in Candida albicans. J Bacteriol. 1996; 178:121-29. https://doi.org/10.1128/jb.178.1.121-129.1996. PMID:8550405 8550405 PMC177628

[114] Papon N, Courdavault V, Lanoue A, Clastre M, Brock M Illuminating fungal infections with bioluminescence. PLoS Pathog. 2014; 10:e1004179.https://doi.org/10.1371/journal.ppat.1004179. PMID:25010008 25010008 PMC4092138

[115] Zhao H, Doyle TC, Wong RJ, Cao Y, Stevenson DK, Piwnica-Worms D, Contag CH Characterization of coelenterazine analogs for measurements of Renilla luciferase activity in live cells and living animals. Mol Imaging. 2004; 3:43-54. https://doi.org/10.1162/153535004773861714. PMID:15142411 15142411

[116] Tannous BA, Kim DE, Fernandez JL, Weissleder R, Breakefield XO Codon-optimized Gaussia luciferase cDNA for mammalian gene expression in culture and *in vivo*. Mol Ther. 2005; 11:435-43. https://doi.org/10.1016/j.ymthe.2004.10.016. PMID:15727940 15727940

[117] Rice BW, Cable MD, Nelson MB In vivo imaging of light-emitting probes. J Biomed Opt. 2001; 6:432-40. https://doi.org/10.1117/1.1413210. PMID:11728202 11728202

[118] Turman MA, Mathews A A simple luciferase assay to measure atp levels in small numbers of cells using a fluorescent plate reader. In Vitro Cell Dev Biol Anim. 1996; 32:1-4. https://doi.org/10.1007/BF02722985. PMID:8835310 8835310

[119] Brock M Application of bioluminescence imaging for *in vivo* monitoring of fungal infections. Int J Microbiol. 2012; 2012:956794.https://doi.org/10.1155/2012/956794. PMID:22121368 22121368 PMC3205719

[120] Tatsumi H, Masuda T, Nakano E Synthesis of Enzymatically Active Firefly Luciferase in Yeast. Agricultural and Biological Chemistry. 1988; 52:1123-27.

[121] Vieites JM, Navarro-García F, Pérez-Díaz R, Pla J, Nombela C Expression and *in vivo* determination of firefly luciferase as gene reporter in Saccharomyces cerevisiae. Yeast. 1994; 10:1321-27. https://doi.org/10.1002/yea.320101009. PMID:7900421 7900421

[122] Leskinen P, Virta M, Karp M One-step measurement of firefly luciferase activity in yeast. Yeast. 2003; 20:1109-13. https://doi.org/10.1002/yea.1024. PMID:14558144 14558144

[123] Suzuki T, Ueda T, Ohama T, Osawa S, Watanabe K The gene for serine tRNA having anticodon sequence CAG in a pathogenic yeast, Candida albicans. Nucleic Acids Res. 1993; 21:356.https://doi.org/10.1093/nar/21.2.356. PMID:8441644 8441644 PMC309117

[124] de Wet JR, Wood KV, DeLuca M, Helinski DR, Subramani S Firefly luciferase gene: structure and expression in mammalian cells. Mol Cell Biol. 1987; 7:725-37. https://doi.org/10.1128/MCB.7.2.725. PMID:3821727 3821727 PMC365129

[125] Doyle TC, Nawotka KA, Purchio AF, Akin AR, Francis KP, Contag PR Expression of firefly luciferase in Candida albicans and its use in the selection of stable transformants. Microb Pathog. 2006; 40:69-81. https://doi.org/10.1016/j.micpath.2005.11.002. PMID:16427765 16427765

[126] d'Enfert C, Vecchiarelli A, Brown AJ Bioluminescent fungi for real-time monitoring of fungal infections. Virulence. 2010; 1:174-76. https://doi.org/10.4161/viru.1.3.11119. PMID:21178436 21178436

[127] Brock M, Jouvion G, Droin-Bergère S, Dussurget O, Nicola MA, Ibrahim-Granet O Bioluminescent Aspergillus fumigatus, a new tool for drug efficiency testing and *in vivo* monitoring of invasive aspergillosis. Appl Environ Microbiol. 2008; 74:7023-35. https://doi.org/10.1128/AEM.01288-08. PMID:18820063 18820063 PMC2583481

[128] Galiger C, Brock M, Jouvion G, Savers A, Parlato M, Ibrahim-Granet O Assessment of efficacy of antifungals against Aspergillus fumigatus: value of real-time bioluminescence imaging. Antimicrob Agents Chemother. 2013; 57:3046-59. https://doi.org/10.1128/AAC.01660-12. PMID:23587947 23587947 PMC3697358

[129] Donat S, Hasenberg M, Schäfer T, Ohlsen K, Gunzer M, Einsele H, Löffler J, Beilhack A, Krappmann S Surface display of Gaussia princeps luciferase allows sensitive fungal pathogen detection during cutaneous aspergillosis. Virulence. 2012; 3:51-61. https://doi.org/10.4161/viru.3.1.18799. PMID:22286700 22286700

[130] Vecchiarelli A, d'Enfert C Shedding natural light on fungal infections. Virulence. 2012; 3:15-17. https://doi.org/10.4161/viru.3.1.19247. PMID:22286695 22286695

[131] Gooch VD, Mehra A, Larrondo LF, Fox J, Touroutoutoudis M, Loros JJ, Dunlap JC Fully codon-optimized luciferase uncovers novel temperature characteristics of the Neurospora clock. Eukaryot Cell. 2008; 7:28-37. https://doi.org/10.1128/EC.00257-07. PMID:17766461 17766461 PMC2224151

[132] Doyle TC, Nawotka KA, Kawahara CB, Francis KP, Contag PR Visualizing fungal infections in living mice using bioluminescent pathogenic Candida albicans strains transformed with the firefly luciferase gene. Microb Pathog. 2006; 40:82-90. https://doi.org/10.1016/j.micpath.2005.11.003. PMID:16426810 16426810

[133] Pietrella D, Rachini A, Torosantucci A, Chiani P, Brown AJ, Bistoni F, Costantino P, Mosci P, d'Enfert C, Rappuoli R, et al. A beta-glucan-conjugate vaccine and anti-beta-glucan antibodies are effective against murine vaginal candidiasis as assessed by a novel *in vivo* imaging technique. Vaccine. 2010; 28:1717-25. https://doi.org/10.1016/j.vaccine.2009.12.021. PMID:20038431 20038431

[134] Vande Velde G, Kucharíková S, Schrevens S, Himmelreich U, Van Dijck P Towards non-invasive monitoring of pathogen-host interactions during Candida albicans biofilm formation using *in vivo* bioluminescence. Cell Microbiol. 2014; 16:115-30. https://doi.org/10.1111/cmi.12184. PMID:23962311 23962311 PMC4204156

[135] Vande Velde G, Kucharíková S, Van Dijck P, Himmelreich U Bioluminescence imaging of fungal biofilm development in live animals. Methods Mol Biol. 2014; 1098:153-67. https://doi.org/10.1007/978-1-62703-718-1_13. PMID:24166376 24166376

[136] Franke-Fayard B, Waters AP, Janse CJ Real-time *in vivo* imaging of transgenic bioluminescent blood stages of rodent malaria parasites in mice. Nat Protoc. 2006; 1:476-85. https://doi.org/10.1038/nprot.2006.69. PMID:17406270 17406270

[137] Braks J, Aime E, Spaccapelo R, Klop O, Janse CJ, Franke-Fayard B Bioluminescence imaging of P. berghei Schizont sequestration in rodents. Methods Mol Biol. 2013; 923:353-68. https://doi.org/10.1007/978-1-62703-026-7_25. PMID:22990791 22990791

[138] Lin JW, Sajid M, Ramesar J, Khan SM, Janse CJ, Franke-Fayard B Screening inhibitors of P. berghei blood stages using bioluminescent reporter parasites. Methods Mol Biol. 2013; 923:507-22. https://doi.org/10.1007/978-1-62703-026-7_35. PMID:22990801 22990801

[139] Ploemen I, Behet M, Nganou-Makamdop K, van Gemert GJ, Bijker E, Hermsen C, Sauerwein R Evaluation of immunity against malaria using luciferase-expressing Plasmodium berghei parasites. Malar J. 2011; 10:350.https://doi.org/10.1186/1475-2875-10-350. PMID:22152047 22152047 PMC3281144

[140] Miller JL, Murray S, Vaughan AM, Harupa A, Sack B, Baldwin M, Crispe IN, Kappe SH Quantitative bioluminescent imaging of pre-erythrocytic malaria parasite infection using luciferase-expressing Plasmodium yoelii. PLoS One. 2013; 8:e60820.https://doi.org/10.1371/journal.pone.0060820. PMID:23593316 23593316 PMC3623966

[141] Li Q, Xie L, Caridha D, Roncal N, Zeng Q, Zhang J, Zhang P, Hickman M, Read L Comparative Susceptibility of Different Mouse Strains to Liver-Stage Infection With Plasmodium berghei Sporozoites Assessed Using In Vivo Imaging. Mil Med. 2017; 182:360-68. https://doi.org/10.7205/MILMED-D-16-00090. PMID:28291500 28291500

[142] Paloque L, Vidal N, Casanova M, Dumètre A, Verhaeghe P, Parzy D, Azas N A new, rapid and sensitive bioluminescence assay for drug screening on Leishmania. J Microbiol Methods. 2013; 95:320-23. https://doi.org/10.1016/j.mimet.2013.09.006. PMID:24055386 24055386

[143] Beattie L, Evans KJ, Kaye PM, Smith DF Transgenic Leishmania and the immune response to infection. Parasite Immunol. 2008; 30:255-66. https://doi.org/10.1111/j.1365-3024.2008.01020.x. PMID:18266814 18266814 PMC3876712

[144] Lang T, Goyard S, Lebastard M, Milon G Bioluminescent Leishmania expressing luciferase for rapid and high throughput screening of drugs acting on amastigote-harbouring macrophages and for quantitative real-time monitoring of parasitism features in living mice. Cell Microbiol. 2005; 7:383-92. https://doi.org/10.1111/j.1462-5822.2004.00468.x. PMID:15679841 15679841

[145] de La Llave E, Lecoeur H, Besse A, Milon G, Prina E, Lang T A combined luciferase imaging and reverse transcription polymerase chain reaction assay for the study of Leishmania amastigote burden and correlated mouse tissue transcript fluctuations. Cell Microbiol. 2011; 13:81-91. https://doi.org/10.1111/j.1462-5822.2010.01521.x. PMID:20846338 20846338

[146] Reimão JQ, Trinconi CT, Yokoyama-Yasunaka JK, Miguel DC, Kalil SP, Uliana SR Parasite burden in Leishmania (Leishmania) amazonensis-infected mice: validation of luciferase as a quantitative tool. J Microbiol Methods. 2013; 93:95-101. https://doi.org/10.1016/j.mimet.2013.02.007. PMID:23466934 23466934

[147] Rouault E, Lecoeur H, Meriem AB, Minoprio P, Goyard S, Lang T Imaging visceral leishmaniasis in real time with golden hamster model: Monitoring the parasite burden and hamster transcripts to further characterize the immunological responses of the host. Parasitol Int. 2017; 66:933-39. https://doi.org/10.1016/j.parint.2016.10.020. PMID:27794505 27794505

[148] Kessler RL, Gradia DF, Pontello Rampazzo Rde C, Lourenço ÉE, Fidêncio NJ, Manhaes L, Probst CM, Ávila AR, Fragoso SP Stage-regulated GFP Expression in Trypanosoma cruzi: applications from host-parasite interactions to drug screening. PLoS One. 2013; 8:e67441. https://doi.org/10.1371/journal.pone.0067441. PMID:23840703 23840703 PMC3688654

[149] D'Archivio S, Cosson A, Medina M, Lang T, Minoprio P, Goyard S Non-invasive *in vivo* study of the Trypanosoma vivax infectious process consolidates the brain commitment in late infections. PLoS Negl Trop Dis. 2013; 7:e1976.https://doi.org/10.1371/journal.pntd.0001976. PMID:23301112 23301112 PMC3536815

[150] Myburgh E, Coles JA, Ritchie R, Kennedy PG, McLatchie AP, Rodgers J, Taylor MC, Barrett MP, Brewer JM, Mottram JC In vivo imaging of trypanosome-brain interactions and development of a rapid screening test for drugs against CNS stage trypanosomiasis. PLoS Negl Trop Dis. 2013; 7:e2384.https://doi.org/10.1371/journal.pntd.0002384. PMID:23991236 23991236 PMC3749981

[151] Martinez-Calvillo S, Lopez I, Hernandez R pRIBOTEX expression vector: a pTEX derivative for a rapid selection of Trypanosoma cruzi transfectants. Gene. 1997; 199:71-76. https://doi.org/10.1016/S0378-1119(97)00348-X. PMID:9358041 9358041

[152] Canavaci AM, Bustamante JM, Padilla AM, Perez Brandan CM, Simpson LJ, Xu D, Boehlke CL, Tarleton RL In vitro and *in vivo* high-throughput assays for the testing of anti-Trypanosoma cruzi compounds. PLoS Negl Trop Dis. 2010; 4:e740.https://doi.org/10.1371/journal.pntd.0000740. PMID:20644616 20644616 PMC2903469

[153] Claes F, Vodnala SK, van Reet N, Boucher N, Lunden-Miguel H, Baltz T, Goddeeris BM, Büscher P, Rottenberg ME Bioluminescent imaging of Trypanosoma brucei shows preferential testis dissemination which may hamper drug efficacy in sleeping sickness. PLoS Negl Trop Dis. 2009; 3:e486.https://doi.org/10.1371/journal.pntd.0000486. PMID:19621071 19621071 PMC2707598

[154] Henriques C, Castro DP, Gomes LH, Garcia ES, de Souza W Bioluminescent imaging of Trypanosoma cruzi infection in Rhodnius prolixus. Parasit Vectors. 2012; 5:214.https://doi.org/10.1186/1756-3305-5-214. PMID:23013827 23013827 PMC3481367

[155] Silva-Dos-Santos D, Barreto-de-Albuquerque J, Guerra B, Moreira OC, Berbert LR, Ramos MT, Mascarenhas BAS, Britto C, Morrot A, Serra Villa-Verde DM, et al. Unraveling Chagas disease transmission through the oral route: Gateways to Trypanosoma cruzi infection and target tissues. PLoS Negl Trop Dis. 2017; 11:e0005507.https://doi.org/10.1371/journal.pntd.0005507. PMID:28379959 28379959 PMC5397068

[156] Dellacasa-Lindberg I, Hitziger N, Barragan A Localized recrudescence of Toxoplasma infections in the central nervous system of immunocompromised mice assessed by *in vivo* bioluminescence imaging. Microbes Infect. 2007; 9:1291-98. https://doi.org/10.1016/j.micinf.2007.06.003. PMID:17897859 17897859

[157] Subauste C Animal models for Toxoplasma gondii infection. Curr Protoc Immunol. 2012;Chapter 19: Unit 19 13:11-23. 10.1002/0471142735.im1903s96 22314833

[158] Kamerkar S, Davis PH Toxoplasma on the brain: understanding host-pathogen interactions in chronic CNS infection. J Parasitol Res. 2012; 2012:589295.https://doi.org/10.1155/2012/589295. PMID:22545203 22545203 PMC3321570

[159] Saeij JP, Boyle JP, Grigg ME, Arrizabalaga G, Boothroyd JC Bioluminescence imaging of Toxoplasma gondii infection in living mice reveals dramatic differences between strains. Infect Immun. 2005; 73:695-702. https://doi.org/10.1128/IAI.73.2.695-702.2005. PMID:15664907 15664907 PMC547072

[160] Hitziger N, Dellacasa I, Albiger B, Barragan A Dissemination of Toxoplasma gondii to immunoprivileged organs and role of Toll/interleukin-1 receptor signalling for host resistance assessed by *in vivo* bioluminescence imaging. Cell Microbiol. 2005; 7:837-48. https://doi.org/10.1111/j.1462-5822.2005.00517.x. PMID:15888086 15888086

[161] Swedin L, Arrighi R, Andersson-Willman B, Murray A, Chen Y, Karlsson MC, Georén SK, Tkach AV, Shvedova AA, Fadeel B, et al. Pulmonary exposure to single-walled carbon nanotubes does not affect the early immune response against Toxoplasma gondii. Part Fibre Toxicol. 2012; 9:16.https://doi.org/10.1186/1743-8977-9-16. PMID:22621311 22621311 PMC3495637

[162] Luker KE, Luker GD Applications of bioluminescence imaging to antiviral research and therapy: multiple luciferase enzymes and quantitation. Antiviral Res. 2008; 78:179-87. https://doi.org/10.1016/j.antiviral.2008.01.158. PMID:18358543 18358543 PMC2430099

[163] Rodriguez JF, Rodriguez D, Rodriguez JR, McGowan EB, Esteban M Expression of the firefly luciferase gene in vaccinia virus: a highly sensitive gene marker to follow virus dissemination in tissues of infected animals. Proc Natl Acad Sci U S A. 1988; 85:1667-71. https://doi.org/10.1073/pnas.85.5.1667. PMID:3422754 3422754 PMC279835

[164] Zaitseva M, Kapnick S, Golding H Measurements of vaccinia virus dissemination using whole body imaging: approaches for predicting of lethality in challenge models and testing of vaccines and antiviral treatments. Methods Mol Biol. 2012; 890:161-76. https://doi.org/10.1007/978-1-61779-876-4_10. PMID:22688767 22688767

[165] Luker KE, Hutchens M, Schultz T, Pekosz A, Luker GD Bioluminescence imaging of vaccinia virus: effects of interferon on viral replication and spread. Virology. 2005; 341:284-300. https://doi.org/10.1016/j.virol.2005.06.049. PMID:16095645 16095645

[166] Lipshutz GS, Gruber CA, Cao Y, Hardy J, Contag CH, Gaensler KM In utero delivery of adeno-associated viral vectors: intraperitoneal gene transfer produces long-term expression. Mol Ther. 2001; 3:284-92. https://doi.org/10.1006/mthe.2001.0267. PMID:11273769 11273769

[167] Luker GD, Prior JL, Song J, Pica CM, Leib DA Bioluminescence imaging reveals systemic dissemination of herpes simplex virus type 1 in the absence of interferon receptors. J Virol. 2003; 77:11082-93. https://doi.org/10.1128/JVI.77.20.11082-11093.2003. PMID:14512556 14512556 PMC224994

[168] Luker GD, Bardill JP, Prior JL, Pica CM, Piwnica-Worms D, Leib DA Noninvasive bioluminescence imaging of herpes simplex virus type 1 infection and therapy in living mice. J Virol. 2002; 76:12149-61. https://doi.org/10.1128/JVI.76.23.12149-12161.2002. PMID:12414955 12414955 PMC136903

[169] Murphy AA, Rosato PC, Parker ZM, Khalenkov A, Leib DA Synergistic control of herpes simplex virus pathogenesis by IRF-3, and IRF-7 revealed through non-invasive bioluminescence imaging. Virology. 2013; 444:71-79. https://doi.org/10.1016/j.virol.2013.05.034. PMID:23777662 23777662 PMC3755050

[170] Cook SH, Griffin DE Luciferase imaging of a neurotropic viral infection in intact animals. J Virol. 2003; 77:5333-38. https://doi.org/10.1128/JVI.77.9.5333-5338.2003. PMID:12692235 12692235 PMC153972

[171] Doyle TC, Burns SM, Contag CH In vivo bioluminescence imaging for integrated studies of infection. Cell Microbiol. 2004; 6:303-17. https://doi.org/10.1111/j.1462-5822.2004.00378.x. PMID:15009023 15009023

[172] Sun C, Gardner CL, Watson AM, Ryman KD, Klimstra WB Stable, high-level expression of reporter proteins from improved alphavirus expression vectors to track replication and dissemination during encephalitic and arthritogenic disease. J Virol. 2014; 88:2035-46. https://doi.org/10.1128/JVI.02990-13. PMID:24307590 24307590 PMC3911548

[173] Li K, Thomasson D, Ketai L, Contag C, Pomper M, Wright M, Bray M Potential applications of conventional and molecular imaging to biodefense research. Clin Infect Dis. 2005; 40:1471-80. https://doi.org/10.1086/429723. PMID:15844070 15844070

[174] Zaitseva M, Kapnick SM, Scott J, King LR, Manischewitz J, Sirota L, Kodihalli S, Golding H Application of bioluminescence imaging to the prediction of lethality in vaccinia virus-infected mice. J Virol. 2009; 83:10437-47. https://doi.org/10.1128/JVI.01296-09. PMID:19656894 19656894 PMC2753125

[175] Americo JL, Sood CL, Cotter CA, Vogel JL, Kristie TM, Moss B, Earl PL Susceptibility of the wild-derived inbred CAST/Ei mouse to infection by orthopoxviruses analyzed by live bioluminescence imaging. Virology. 2014; 449:120-32. https://doi.org/10.1016/j.virol.2013.11.017. PMID:24418545 24418545 PMC3902144

[176] Luker KE, Schultz T, Romine J, Leib DA, Luker GD Transgenic reporter mouse for bioluminescence imaging of herpes simplex virus 1 infection in living mice. Virology. 2006; 347:286-295. https://doi.org/10.1016/j.virol.2005.12.016. PMID:16430938 16430938

[177] Burgos JS, Guzman-Sanchez F, Sastre I, Fillat C, Valdivieso F Non-invasive bioluminescence imaging for monitoring herpes simplex virus type 1 hematogenous infection. Microbes Infect. 2006; 8:1330-38. https://doi.org/10.1016/j.micinf.2005.12.021. PMID:16682243 16682243

[178] Wang L, Fu Q, Dong Y, Zhou Y, Jia S, Du J, Zhao F, Wang Y, Wang X, Peng J, Yang S, et al. Bioluminescence imaging of Hepatitis C virus NS3/4A serine protease activity in cells and living animals. Antiviral Res. 2010; 87:50-56. https://doi.org/10.1016/j.antiviral.2010.04.010. PMID:20420854 20420854

[179] Close DM, Xu T, Sayler GS, Ripp S In vivo bioluminescent imaging (BLI): noninvasive visualization and interrogation of biological processes in living animals. Sensors (Basel). 2011; 11:180-206. https://doi.org/10.3390/s110100180. PMID:22346573 22346573 PMC3274065

[180] Du J, Zhao F, Zhou Y, Yan H, Duan XG, Liang SQ, Wang YL, Fu QX, Wang XH, Peng JC, et al. Bioluminescence imaging allows monitoring hepatitis C virus core protein inhibitors in mice. PLoS One. 2010; 5:e14043.https://doi.org/10.1371/journal.pone.0014043. PMID:21124971 21124971 PMC2987796

[181] Billerbeck E, Horwitz JA, Labitt RN, Donovan BM, Vega K, Budell WC, Koo GC, Rice CM, Ploss A Characterization of human antiviral adaptive immune responses during hepatotropic virus infection in HLA-transgenic human immune system mice. J Immunol. 2013; 191:1753-64. https://doi.org/10.4049/jimmunol.1201518. PMID:23833235 23833235 PMC3735836

[182] Du J, Zhou Y, Fu QX, Gong WL, Zhao F, Peng JC, Zhan LS Bioluminescence imaging of hepatitis B virus enhancer and promoter activities in mice. FEBS Lett. 2008; 582:3552-56. https://doi.org/10.1016/j.febslet.2008.09.035. PMID:18822287 18822287

[183] Zhao F, Liang SQ, Zhou Y, Wang YL, Yan H, Wang XH, Wang HP, Du J, Zhan LS Evaluation of hepatitis B virus promoters for sustained transgene expression in mice by bioluminescence imaging. Virus Res. 2010; 149:162-66. https://doi.org/10.1016/j.virusres.2010.01.012. PMID:20122974 20122974

[184] Liang SQ, Du J, Yan H, Zhou QQ, Zhou Y, Yuan ZN, Yan SD, Fu QX, Wang XH, Jia SZ, et al. A mouse model for studying the clearance of hepatitis B virus *in vivo* using a luciferase reporter. PLoS One. 2013; 8:e60005.https://doi.org/10.1371/journal.pone.0060005. PMID:23577080 23577080 PMC3618179

[185] Harmache A, LeBerre M, Droineau S, Giovannini M, Brémont M Bioluminescence imaging of live infected salmonids reveals that the fin bases are the major portal of entry for Novirhabdovirus. J Virol. 2006; 80:3655-59. https://doi.org/10.1128/JVI.80.7.3655-3659.2006. PMID:16537634 16537634 PMC1440417

[186] Li XF, Li XD, Deng CL, Dong HL, Zhang QY, Ye Q, Ye HQ, Huang XY, Deng YQ, Zhang B, et al. Visualization of a neurotropic flavivirus infection in mouse reveals unique viscerotropism controlled by host type I interferon signaling. Theranostics. 2017; 7:912-25. https://doi.org/10.7150/thno.16615. PMID:28382163 28382163 PMC5381253

[187] Karlsson EA, Meliopoulos VA, Savage C, Livingston B, Mehle A, Schultz-Cherry S Visualizing real-time influenza virus infection, transmission and protection in ferrets. Nat Commun. 2015; 6:6378.https://doi.org/10.1038/ncomms7378. PMID:25744559 25744559 PMC4366512

[188] Luker KE, Luker GD Bioluminescence imaging of reporter mice for studies of infection and inflammation. Antiviral Res. 2010; 86:93-100. https://doi.org/10.1016/j.antiviral.2010.02.002. PMID:20417377 20417377 PMC2863000

[189] Karsi A, Howe K, Kirkpatrick TB, Wills R, Bailey RH, Lawrence ML Development of bioluminescent Salmonella strains for use in food safety. BMC Microbiol. 2008; 8:10.https://doi.org/10.1186/1471-2180-8-10. PMID:18211715 18211715 PMC2257966

[190] Kassem II, Sanad Y, Gangaiah D, Lilburn M, Lejeune J, Rajashekara G Use of bioluminescence imaging to monitor Campylobacter survival in chicken litter. J Appl Microbiol. 2010; 109:1988-997. https://doi.org/10.1111/j.1365-2672.2010.04828.x. PMID:20722878 20722878

[191] Xu X, Miller SA, Baysal-Gurel F, Gartemann KH, Eichenlaub R, Rajashekara G Bioluminescence imaging of Clavibacter michiganensis subsp. michiganensis infection of tomato seeds and plants. Appl Environ Microbiol. 2010; 76:3978-88. https://doi.org/10.1128/AEM.00493-10. PMID:20400561 20400561 PMC2893490

[192] Kassem II, Splitter GA, Miller S, Rajashekara G Let There Be Light! Bioluminescent Imaging to Study Bacterial Pathogenesis in Live Animals and Plants. Adv Biochem Eng Biotechnol. 2014; 154: 119–45. https://doi:10.1007/10_2014_280 PMID: 25395174 25395174

[193] Chaudhari AJ, Darvas F, Bading JR, Moats RA, Conti PS, Smith DJ, Cherry SR, Leahy RM Hyperspectral and multispectral bioluminescence optical tomography for small animal imaging. Phys Med Biol. 2005; 50:5421-41. https://doi.org/10.1088/0031-9155/50/23/001. PMID:16306643 16306643

[194] Collins JW, Meganck JA, Kuo C, Francis KP, Frankel G 4D multimodality imaging of Citrobacter rodentium infections in mice. J Vis Exp. 2013; 78: 50450 https://doi.org/ 10.3791/50450 PMID: 23979310 PMC3855914 23979310

[195] Laxminarayan R, Powers JH Antibacterial R&D incentives. Nat Rev Drug Discov. 2011; 10:727-28. https://doi.org/10.1038/nrd3560. PMID:21959280 21959280

[196] Cooper MA, Shlaes D Fix the antibiotics pipeline. Nature. 2011; 472:32.https://doi.org/10.1038/472032a. PMID:21475175 21475175

[197] Shlaes DM The abandonment of antibacterials: why and wherefore? Curr Opin Pharmacol. 2003; 3:470-73. https://doi.org/10.1016/j.coph.2003.04.003. PMID:14559090 14559090

[198] O'Neill J Review on Antimicrobial Resistance. Antimicrobial Resistance: Tackling a Crisis for the Health and Wealth of Nations. 2014 pp. http://amr-review.org/sites/default/files/AMR%20Review%20Paper%20-%20Tackling%20a%20crisis%20for%20the%20health%20and%20wealth%20of%20nations_21.pdf.

[199] Thorne N, Inglese J, Auld DS Illuminating insights into firefly luciferase and other bioluminescent reporters used in chemical biology. Chem Biol. 2010; 17:646-57. https://doi.org/10.1016/j.chembiol.2010.05.012. PMID:20609414 20609414 PMC2925662

[200] Feder JL, Velez S Intergenic exchange, geographic isolation, and the evolution of bioluminescent color for Pyrophorus click beetles. Evolution. 2009; 63:1203-16. https://doi.org/10.1111/j.1558-5646.2009.00623.x. PMID:19154393 19154393

[201] Nishiguchi T, Yamada T, Nasu Y, Ito M, Yoshimura H, Ozawa T Development of red-shifted mutants derived from luciferase of Brazilian click beetle Pyrearinus termitilluminans. J Biomed Opt. 2015; 20:101205.https://doi.org/10.1117/1.JBO.20.10.101205. PMID:26313214 26313214

[202] Bhaumik S, Gambhir SS Optical imaging of Renilla luciferase reporter gene expression in living mice. Proc Natl Acad Sci U S A. 2002; 99:377-82. https://doi.org/10.1073/pnas.012611099. PMID:11752410 11752410 PMC117568

[203] Kim SB, Izumi H Functional artificial luciferases as an optical readout for bioassays. Biochem Biophys Res Commun. 2014; 448:418-23. https://doi.org/10.1016/j.bbrc.2014.04.128. PMID:24802399 24802399

[204] Chopra A. Gaussia princeps luciferase. Molecular Imaging and Contrast Agent Database (MICAD). Bethesda (MD); 2004-2013 PMID: 20641352

[205] Francis KP, Joh D, Bellinger-Kawahara C, Hawkinson MJ, Purchio TF, Contag PR Monitoring bioluminescent Staphylococcus aureus infections in living mice using a novel luxABCDE construct. Infect Immun. 2000; 68:3594-600. https://doi.org/10.1128/IAI.68.6.3594-3600.2000. PMID:10816517 10816517 PMC97648

[206] Golberg A, Broelsch GF, Vecchio D, Khan S, Hamblin MR, Austen WG Jr, Sheridan RL, Yarmush ML Eradication of multidrug-resistant A. baumannii in burn wounds by antiseptic pulsed electric field. Technology (Singap World Sci). 2014; 2:153-60. PMID:25089285 25089285 PMC4117356

[207] Huang L, Wang M, Dai T, Sperandio FF, Huang YY, Xuan Y, Chiang LY, Hamblin MR Antimicrobial photodynamic therapy with decacationic monoadducts and bisadducts of [70]fullerene: *in vitro* and *in vivo* studies. Nanomedicine (Lond). 2014; 9:253-66. https://doi.org/10.2217/nnm.13.22. PMID:23738632 23738632 PMC3859801

[208] Dai T, Gupta A, Huang YY, Yin R, Murray CK, Vrahas MS, Sherwood ME, Tegos GP, Hamblin MR Blue light rescues mice from potentially fatal Pseudomonas aeruginosa burn infection: efficacy, safety, and mechanism of action. Antimicrob Agents Chemother. 2013; 57:1238-45. https://doi.org/10.1128/AAC.01652-12. PMID:23262998 23262998 PMC3591931

[209] Dai T, Kharkwal GB, Zhao J, St Denis TG, Wu Q, Xia Y, Huang L, Sharma SK, d'Enfert C, Hamblin MR Ultraviolet-C light for treatment of Candida albicans burn infection in mice. Photochem Photobiol. 2011; 87:342-49. https://doi.org/10.1111/j.1751-1097.2011.00886.x. PMID:21208209 21208209 PMC3048910

[210] Lambrechts SA, Demidova TN, Aalders MC, Hasan T, Hamblin MR Photodynamic therapy for Staphylococcus aureus infected burn wounds in mice. Photochem Photobiol Sci. 2005; 4:503-09. https://doi.org/10.1039/b502125a. PMID:15986057 15986057 PMC3071043

[211] Dai T, Murray CK, Vrahas MS, Baer DG, Tegos GP, Hamblin MR Ultraviolet C light for Acinetobacter baumannii wound infections in mice: potential use for battlefield wound decontamination? J Trauma Acute Care Surg. 2012; 73:661-67. https://doi.org/10.1097/TA.0b013e31825c149c. PMID:22929495 22929495 PMC3463377

[212] Hu Y, Hegde V, Johansen D, Loftin AH, Dworsky E, Zoller SD, Park HY, Hamad CD, Nelson GE, Francis KP, et al. Combinatory antibiotic therapy increases rate of bacterial kill but not final outcome in a novel mouse model of Staphylococcus aureus spinal implant infection. PLoS One. 2017; 12:e0173019.https://doi.org/10.1371/journal.pone.0173019. PMID:28245229 28245229 PMC5330510

[213] van Zyl WF, Deane SM, Dicks LM Enterococcus mundtii ST4SA and Lactobacillus plantarum 423 excludes Listeria monocytogenes from the GIT, as shown by bioluminescent studies in mice. Benef Microbes. 2016; 7:227-35. https://doi.org/10.3920/BM2015.0082. PMID:26689230 26689230

[214] Witcomb LA, Collins JW, McCarthy AJ, Frankel G, Taylor PW Bioluminescent imaging reveals novel patterns of colonization and invasion in systemic Escherichia coli K1 experimental infection in the neonatal rat. Infect Immun. 2015; 83:4528-40. https://doi.org/10.1128/IAI.00953-15. PMID:26351276 26351276 PMC4645386

[215] Ghoneim HE, McCullers JA Adjunctive corticosteroid therapy improves lung immunopathology and survival during severe secondary pneumococcal pneumonia in mice. J Infect Dis. 2014; 209:1459-68. https://doi.org/10.1093/infdis/jit653. PMID:24273183 24273183

[216] Munder A, Wölbeling F, Klockgether J, Wiehlmann L, Tümmler B In vivo imaging of bioluminescent Pseudomonas aeruginosa in an acute murine airway infection model. Pathog Dis. 2014; 72:74-77. https://doi.org/10.1111/2049-632X.12184. PMID:24833236 24833236

[217] Johnson AW, Sidman JD, Lin J Bioluminescent imaging of pneumococcal otitis media in chinchillas. Ann Otol Rhinol Laryngol. 2013; 122:344-52. https://doi.org/10.1177/000348941312200510. PMID:23815053 23815053

[218] Wang X, Li Z, Dong X, Chi H, Wang G, Li J, Sun R, Chen M, Zhang X, Wang Y, et al. Development of Bioluminescent Cronobacter sakazakii ATCC 29544 in a Mouse Model. J Food Prot. 2015; 78:1007-12. https://doi.org/10.4315/0362-028X.JFP-14-482. PMID:25951398 25951398

[219] Kadurugamuwa JL, Sin L, Albert E, Yu J, Francis K, DeBoer M, Rubin M, Bellinger-Kawahara C, Parr TR Jr, Contag PR Direct continuous method for monitoring biofilm infection in a mouse model. Infect Immun. 2003; 71:882-90. https://doi.org/10.1128/IAI.71.2.882-890.2003. PMID:12540570 12540570 PMC145362

[220] Vuong C, Kocianova S, Yu J, Kadurugamuwa JL, Otto M Development of real-time *in vivo* imaging of device-related Staphylococcus epidermidis infection in mice and influence of animal immune status on susceptibility to infection. J Infect Dis. 2008; 198:258-61. https://doi.org/10.1086/589307. PMID:18491976 18491976 PMC2763270

[221] Bayer AS, Abdelhady W, Li L, Gonzales R, Xiong YQ Comparative Efficacies of Tedizolid Phosphate, Linezolid, and Vancomycin in a Murine Model of Subcutaneous Catheter-Related Biofilm Infection Due to Methicillin-Susceptible and -Resistant Staphylococcus aureus. Antimicrob Agents Chemother. 2016; 60:5092-96. https://doi.org/10.1128/AAC.00880-16. PMID:27297485 27297485 PMC4958216

[222] Delarze E, Ischer F, Sanglard D, Coste AT Adaptation of a Gaussia princeps Luciferase reporter system in Candida albicans for *in vivo* detection in the Galleria mellonella infection model. Virulence. 2015; 6:684-93. https://doi.org/10.1080/21505594.2015.1081330. PMID:26305489 26305489 PMC4720271

[223] Slesiona S, Ibrahim-Granet O, Olias P, Brock M, Jacobsen ID Murine infection models for Aspergillus terreus pulmonary aspergillosis reveal long-term persistence of conidia and liver degeneration. J Infect Dis. 2012; 205:1268-77. https://doi.org/10.1093/infdis/jis193. PMID:22438397 22438397

[224] Lewis MD, Fortes Francisco A, Taylor MC, Burrell-Saward H, McLatchie AP, Miles MA, Kelly JM Bioluminescence imaging of chronic Trypanosoma cruzi infections reveals tissue-specific parasite dynamics and heart disease in the absence of locally persistent infection. Cell Microbiol. 2014; 16:1285-300. https://doi.org/10.1111/cmi.12297. PMID:24712539 24712539 PMC4190689

[225] Biteau N, Asencio C, Izotte J, Rousseau B, Fèvre M, Pillay D, Baltz T Trypanosoma brucei gambiense Infections in Mice Lead to Tropism to the Reproductive Organs, and Horizontal and Vertical Transmission. PLoS Negl Trop Dis. 2016; 10:e0004350.https://doi.org/10.1371/journal.pntd.0004350. PMID:26735855 26735855 PMC4703293

[226] Thalhofer CJ, Graff JW, Love-Homan L, Hickerson SM, Craft N, Beverley SM, Wilson ME In vivo imaging of transgenic Leishmania parasites in a live host. J Vis Exp. 2010; 41:1980. https://doi.org/10.3791/1980.PMID:20689512 20689512 PMC3156083

[227] Giraud E, Lecoeur H, Rouault E, Goyard S, Milon G, Lang T A combined luciferase-expressing Leishmania imaging/RT-qPCR assay provides new insights into the sequential bilateral processes deployed in the ear pinna of C57BL/6 mice. Parasitol Int. 2014; 63:245-53. https://doi.org/10.1016/j.parint.2013.08.013. PMID:24001683 24001683

